# Selected strains of the *Ganoderma lucidum* complex from Finnish forests have excellent broadly acting antiviral properties

**DOI:** 10.1038/s41598-025-08377-5

**Published:** 2025-07-02

**Authors:** Dhanik Reshamwala, Sailee Shroff, Jaana Liimatainen, Jenni Tienaho, Ilari Kuukkanen, Mira Laajala, Andrea Civra, Rachele Francese, Pyry Veteli, Marta Cortina-Escribano, Tytti Sarjala, Maarit Karonen, David Lembo, Riikka Linnakoski, Varpu Marjomäki

**Affiliations:** 1 Department of Biological and Environmental Science/Nanoscience Center, University of Jyväskylä, Survontie 9C, Jyväskylä, FI-40014 Finland; 2 Natural Resources Institute Finland (Luke), Helsinki, FI-00790 Finland; 3 Natural Chemistry Research Group, Department of Chemistry, University of Turku, Turku, FI-20014 Finland; 4 Department of Clinical and Biological Sciences, University of Turin, Turin, I-10043 Italy; 5 Natural Resources Institute Finland (Luke), Joensuu, FI-80130 Finland

**Keywords:** Antimicrobial, Broad-spectrum, Stabilization, Enteroviruses, *Ganoderma* sp. ferment, Coronaviruses, Antimicrobials, Bacteria, Cellular microbiology, Fungi, Virology, Biochemistry, Biotechnology, Cell biology, Drug discovery, Microbiology

## Abstract

Broadly acting antivirals are needed to complement vaccines in present and future pandemics and outbreaks as safe and sustainable tools for combating virus infections. Thus, the current study aims to investigate the broad-spectrum antiviral activity of ferments of selected strains belonging to the *Ganoderma lucidum* complex isolated from Finland and investigate their mechanism of action. Cytopathic effect inhibition assay and endpoint assay were used to determine the antiviral activity. The antibacterial activity ws examined using recombinant biosensor strains. Mechanism of action studies included time of addition assay, transmission electron microscopy, confocal microscopy, sucrose gradient separation assay, and thermal assay. The metabolite composition of the ferments was studied using UHPLC-HR-MS/MS. Ferments showed antiviral efficacy against non-enveloped enteroviruses, already at room temperature and within one minute. Broad antimicrobial activity was demonstrated with non-enveloped rotaviruses, enveloped coronaviruses, zika viruses, and gram-positive and gram-negative bacteria. Ferments also directly affected the viruses and caused clustering of the virus particles. Additionally, treatment of enteroviruses with ferments caused strong stabilization of the virus capsid, thus preventing their genome release. UHPLC-HR-MS/MS analysis of the ferments verified the presence of several terpenoid compounds. The results show great promise for the future use of ferments from the *Ganoderma lucidum* complex in combating microbial infections for various applications.

**Supplementary Information:**

The online version contains supplementary material available at 10.1038/s41598-025-08377-5.

## Introduction

In addition to antimicrobial resistance, viruses are one of the biggest threats to world population^1^. In the past 20 years, the most prominent viral outbreaks include severe acute respiratory syndrome coronavirus (SARS-CoV) in 2002–2003 in China, swine flu pandemic (influenza virus A/H1N1) in 2009, the Middle east respiratory syndrome (MERS) coronavirus outbreak in 2012 in the middle east, the West Africa Ebola virus epidemic in 2013–2015, the Zika virus pandemic in 2016–2017, and now, the pandemic caused by SARS-CoV-2. Although not as deadly, enteroviruses are one of the most common pathogens infecting humans, especially young children, elderly people, and immunocompromised individuals. In the US, these viruses are responsible for an estimated 10–15 million cases of symptomatic infections annually^2^. Enteroviruses are responsible for causing acute infections such as meningitis, pancreatitis, myocarditis, and flu^3,4^. They also contribute to chronic infections such as type I diabetes in children, and are also associated with causing exacerbation of asthma and chronic obstructive pulmonary disease (COPD)^5,6^.

Vaccines and antiviral drugs are important for fighting these viral infections. The currently available vaccines are self-limiting as they are effective only against a specific virus they were designed for. Moreover, the process of their development is time consuming and expensive. Logistic and storage obstacles add to the limitations of vaccines as well. The major drawback of the traditional antiviral drugs targeting the host factors is that they cause adverse side effects to the host^7^. In addition, the ones targeting the virus lifecycle may lead to the development of resistance against them due to the high mutation rate of viruses^8^. Previously, the focus was on “one drug-one virus” approach. However, with the need to combat several emerging and seasonal infections, the paradigm has shifted towards “one drug-multiple viruses”^9^. There is a need of developing broad-spectrum antivirals, which can help reduce the viral load or control the viral outbreaks more effectively.

Natural products serve as a rich source of bioactive compounds and have played a significant role in drug discovery and development^10^. They offer structural diversity and have undergone evolutionary selection, optimizing them for interactions with biological macromolecules^11^. Of the 1881 approved drugs between January 1981 to the end of September 2019, total of 41.8% were either biological macromolecules, unaltered natural products, botanical drugs, or natural product derivatives^10^. If the synthetic products mimicking natural compounds are considered, then the share rises to 64.3%^10^. Of the natural products, bioactive compounds of fungal origin have been particularly important and interesting as their production has the potential to be upscaled. The antiviral activity of fungi has been reported to be associated with the high molecular weight compounds (polysaccharides) and small organic molecules (secondary metabolites) present in the mycelium and in the fruiting bodies, as well as in the exudates released in the medium in which the fungus is growing or cultivated^12^. Fungal species belonging to the *Ganoderma* genus have been considered as a particularly rich source of various biologically active compounds^13^ which have been tested in vitro assays^14–17^and in vivo animal experiments^15,18^. In addition to cultivation conditions and bioprocessing techniques^19,20^fungal genotype can also have significant effect on the chemical composition and concentration of bioactive compounds^21,22^. Most of the studies investigating the potential of *Ganoderma* spp. in terms of antiviral, antibacterial, and other bioactive properties have been focused strongly on species and strains of Asian origin. In Finland, the antiviral potential of native *Ganoderma* species has not been studied before. Prompted by the increasing popularity of using *Ganoderma* spp. derived products worldwide, investigations of the antiviral and other biological activities of these fungi existing in other geographical regions are urgently needed.

This study demonstrates that the submerged ferments of strains belonging to the *Ganoderma lucidum* complex originating from Finland have a strong antiviral activity against both non-enveloped and enveloped viruses and, additionally, antibacterial activity against both gram-positive and gram-negative strains. Selected strains with especially high antiviral activity were used to elucidate the antiviral mechanisms of their action.

## Results

### Antiviral screening

**Fig. 1:**
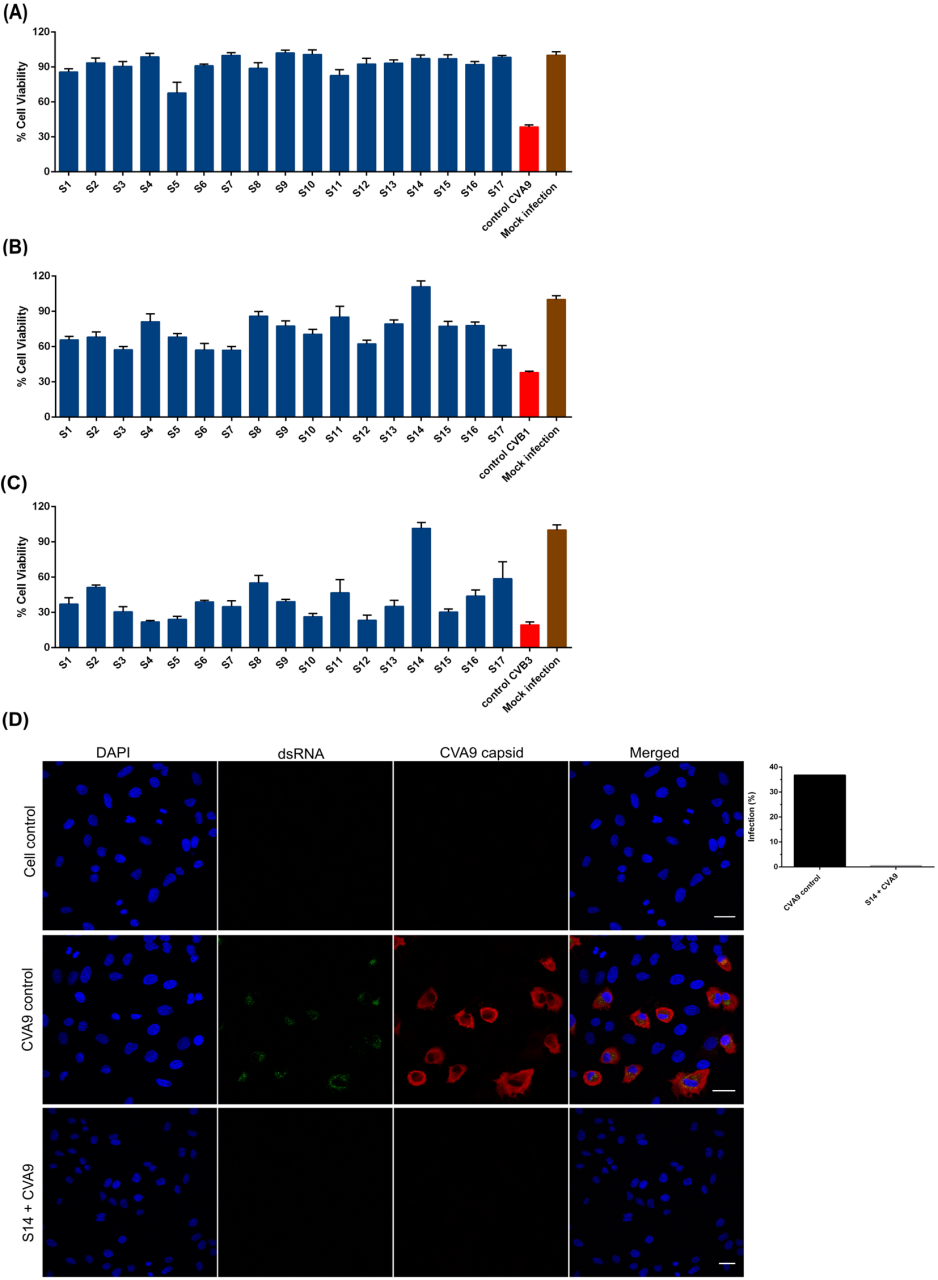
Testing the antiviral activity of ferments of different strains (S1-S17) of *Ganoderma* sp. against enteroviruses. Screening of ferments against (**A**) CVA9 (**B**) CVB1 and (**C**) CVB3, by incubating the ferment-virus mix at 37 °C for 1 h using cytopathic effect (CPE) inhibition assay in A549 cells. 10% v/v of the virus (CVB3, CVB1 and CVA9 titer in the virus-*Ganoderma* sp. ferment mix was 7 × 10^6^ PFU/mL, 5 × 10^6^ PFU/mL, and 3 × 10^8^ PFU/mL, respectively) was pre-treated with the 90% v/v of the ferment. The test samples and the virus control are normalized against the mock infection. Results shown are expressed as average values of three to five technical repeats + standard error of mean from one experiment. (**D**) Studying the effect of ferment on CVA9 using confocal microscopy. Virus was treated with the ferment S14 for 1 h at 37 ℃ before infecting the A549 cells for 6 h. Viral capsid protein (red) and dsRNA (green) were immunolabeled and nucleus was stained with DAPI. The relative number of infected cells was quantified out of all cells, from over 600 cells and plotted. Scale bar 30 μm.

To evaluate the antiviral activity of the different strains of *Ganoderma* sp. belonging to the *G. lucidum* complex, we first screened all the ferments against Coxsackievirus A9 strain (CVA9) using the cytopathic effect inhibition (CPE) assay. In the screening experiment, the ferments (90% v/v) were incubated with high amount of CVA9 (3 × 10^8^ particle forming units (PFU) /mL) for 1 h at 37 °C before adding the affected virus on the cells. Mock infection and untreated virus were used as negative and positive controls, respectively. The screening results showed that all the ferments tested could potently inhibit the virus infection and protect the A549 cells (Fig. 1A). We also tested if the ferments were effective against other enterovirus serotypes. Therefore, we tested them against high amounts of Coxsackievirus B1 and B3 (CVB1, 5 × 10^6^ PFU/mL; CVB3, 7 × 10^6^ PFU/mL) in a similar screening experiment as described for CVA9. All the ferments were effective in inhibiting CVB1 infection (Fig. 1B). From the screening results (Fig. 1C), it was evident that the ferments of strains 2, 8, 11 and 17 were showing moderate antiviral activity against CVB3, while strain 14 showed excellent antiviral activity. The fact that ferments were far more effective against other virus serotypes than CVB3 suggest that the hydrophobic pocket of enteroviruses might be a target of the ferments. Namely, the Nancy strain of CVB3 has a collapsed pocket while CVB1 and CVA9 have a wider pocket^23^.

Based on the screening results, ferments of strains 8, 11, 14 and 17 were selected for further investigations as they seemed to inhibit different serotypes of enteroviruses. Cytotoxicity for these chosen ferments and controls were also tested in a similar manner as described above. The assay showed that none of the selected ferments were toxic to the A549 cells and therefore, the CPE data shows reliable results of the antiviral efficacy (Supplementary Fig.S1). Furthermore, the control medium, where ferments were produced, had no antiviral activity against CVA9 (Supplementary Fig. S2).

Next, we wanted to observe the antiviral effect of the ferments after only one infection cycle. This was studied using confocal microscopy, where the viral capsid protein VP1 as well as the replication intermediate, dsRNA, were immunolabeled and nucleus of cells was stained with 4’,6-diamidino-2-phenylindole (DAPI). CVA9 was first treated with the ferment of strain 8 or 14 for 1 h at 37 ℃ after which the infection was followed on A549 cells for 6 h. No virus was detected in the mock infection, which was expected, and the viral protein antibody did not give any significant background fluorescence. The infection was proven by the cells showing cytoplasm full of newly synthetized VP1 protein and the replication intermediate dsRNA clearly visible in cytoplasmic vesicles (Fig. 1D). The control infection, counted as the number of VP1 positive cells out of all cells labeled with nuclei stain, showed 37% of A549 cells positive for VP1 (calculated from 600 cells). In contrast, protein production or replication was not observed in case of virus treated with the ferment as signal from either VP1 protein or dsRNA was not detected, confirming no infection of the A549 cells (calculated from 600 cells) (Fig. 1D).

### Detailed antiviral efficacy studies

**Fig. 2:**
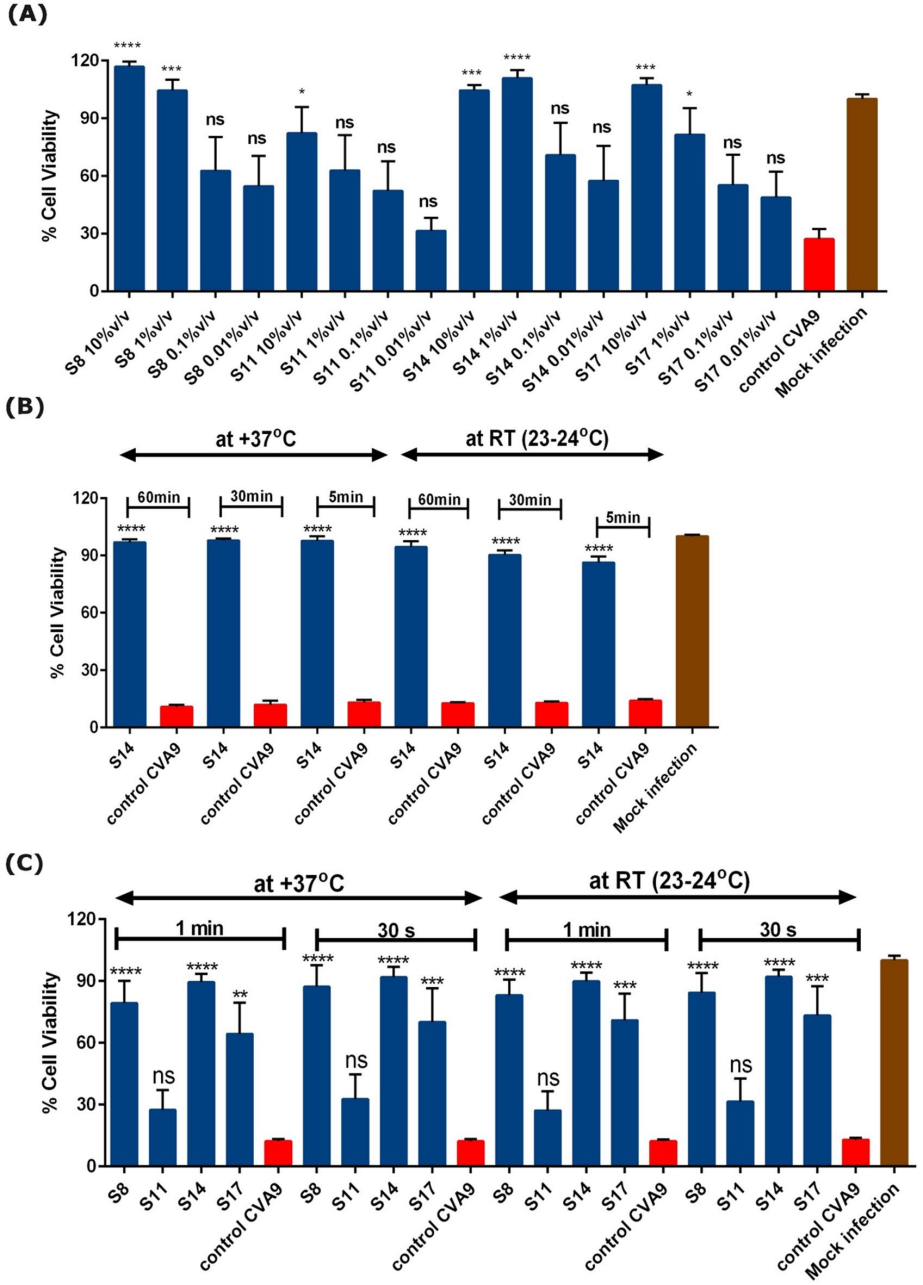
Impact of dilution, time and temperature on the antiviral efficacy of selected strain ferments of *Ganoderma* sp. against CVA9 using cytopathic effect (CPE) inhibition assay in A549 cells (**A**) Different dilutions of ferments were tested, and the virus-ferment mix was incubated at 37 °C for 1 h (**B-C**) The ferment-virus mix was incubated at different time (60-, 30-, 5- and 1-min and 30 s) and temperature (37 °C and room temperature (RT)). The test samples and the virus control are normalized against the mock infection. Experiments A-C were performed twice independently with 2–3 technical repeats per sample. Result expressed as average values + standard error of mean is shown. Statistical significance of the test samples against the virus control was calculated by performing one-way ANOVA, followed by Bonferroni test (*p < 0.05, ***p < 0.001 and ****p < 0.0001). ns = not significant.

To evaluate the antiviral efficacy in more detail, we performed a dilution series experiment. Here, we treated the virus (CVA9 titer in the virus-sample mix was 2 × 10^6^ PFU/mL) with different concentrations (ten-fold serial dilutions) of the ferments for 1 h at 37 °C before adding it on the cells. From the Fig. 2A, it was clear that strain 8, 14 and 17 ferments exhibited good antiviral efficacies when they were diluted 10-fold (10% v/v), whereas strain 11 ferment was less effective. In addition, strain 8 and 14 ferments were equally effective in protecting the cells from CVA9 infection even when they were diluted further 100-fold (1% v/v).

Once the dilution series experiment confirmed that even lower concentrations (10% v/v) of the ferments were equally effective in inhibiting CVA9 infection as the higher concentrations (90% v/v), we performed an end-point dilution assay to quantify the reduction in the virus infectivity of the ferment treated virus. For this, we treated CVA9 with ferment of strain 14 (10% v/v) for 1 h at 37 °C and then made ten-fold serial dilutions of the ferment-virus mix on the cells. Serial dilution of the virus without the ferment was used as a control in the assay. The results showed that there was a 3-log reduction in the virus infectivity when CVA9 was treated with ferment of strain 14 compared to the virus control (Table 1).

**Table 1:** Quantification of the reduction of CVA9 infectivity in A549 cells using endpoint Dilution assay.

Sample Information	Virus titer (PFU/mL)
Virus control	4.13 × 10^10^
S14	3.87 × 10^7^

The virus was treated with strain 14 ferment for 1 h at 37 °C which led to a 3-log reduction in virus titer compared to the virus control. The data is from two independent experiments.

Next, we wanted to evaluate the effect of contact time and temperature on the antiviral activity of the ferment. For this, we pre-treated the virus (CVA9; 3 × 10^8^ PFU/mL) with strain 14 ferment (90% v/v) at different time-points (60 min, 30 min and 5 min) and temperatures (37 °C and room temperature (RT)), before adding it on the cells. When the ferment and virus were incubated at 37 °C for 60 min, strain 14 ferment showed complete antiviral activity as expected (Fig. 2B). Even with the reduction in the incubation time-interval to 5 min, we observed no reduction in the antiviral activity of strain 14. Interestingly, the antiviral activity was retained even when the virus was incubated at RT (23–24 °C) for 5 min with strain 14 ferment as only a small decline in efficacy was observed. We further reduced the incubation time-interval (1 min and 30 s) and tested more of the *Ganoderma* strains (selected based on screening results). Surprisingly, all the strains ferment (except for S11) were effective at inhibiting the viral infection, within just 30 s of incubation at RT (Fig. 2C). Furthermore, we found out that the *Ganoderma* sp. ferment that were stored either at -20 °C for 4 years or at 4 °C for 1–3 years showed excellent antiviral efficacy (Supplementary Fig. S3). The results altogether thus suggest that the selected strains that have a high antiviral activity, were immediately effective at different temperatures and retain their efficacy even after long storage time.

### Mechanism of action

**Fig. 3:**
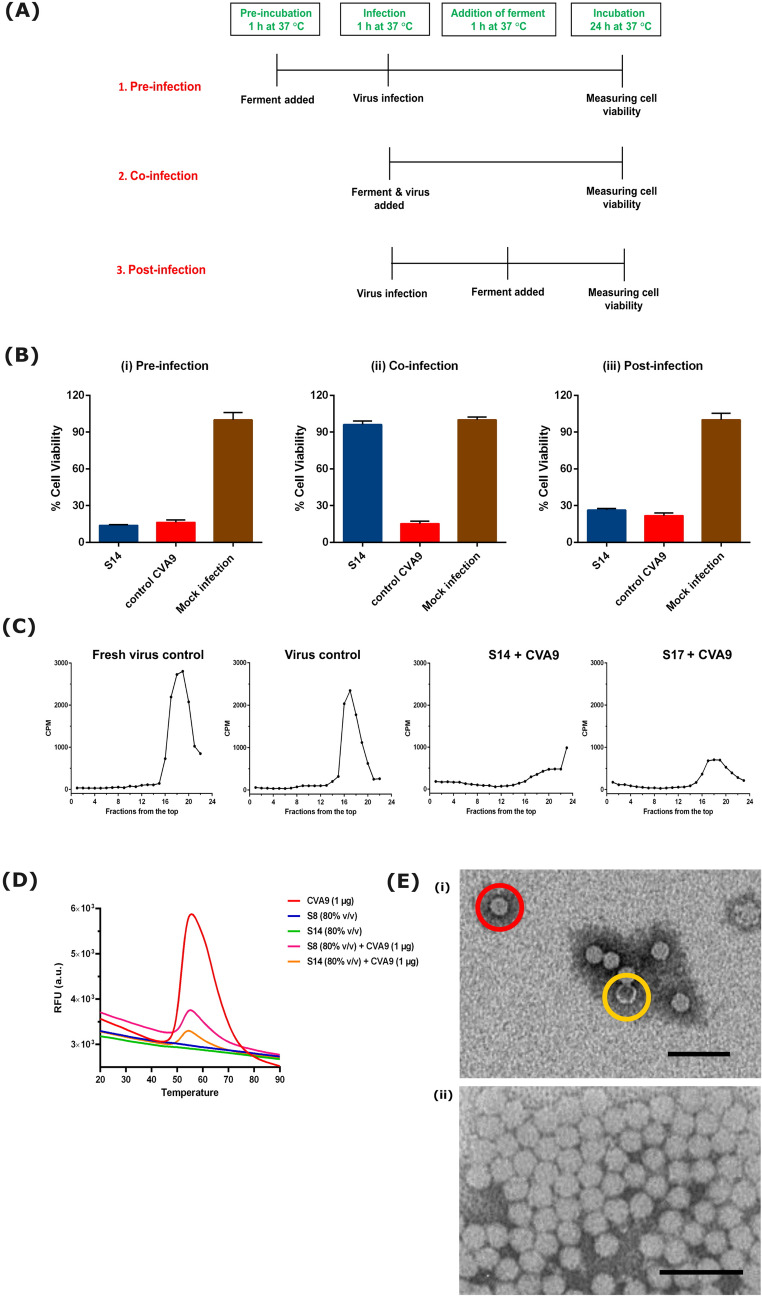
Studies to understand the ferment’s antiviral mechanism of action. (**A**) Schematic of time of addition studies. (**B**) Three modes of infection were studied during time of addition experiment. (i) Pre-infection mode, where the strain 14 ferment was added on cells 1 h before virus infection. (ii) Co-infection mode, where virus and the strain 14 ferment were added directly on cells. (iii) Post-infection mode, where the strain 14 ferment was added on cells 1 h post-infection. Experiment was performed twice independently. Result is expressed as average values + standard error of mean. (**C**) Sucrose gradient (5–20%) separation of the metabolically labeled CVA9 after incubation with the *Ganoderma* sp. ferment for 1 h at 37 °C (**D**) PaSTRy assay used to study the stability of the virus (CVA9) when treated with different ferments. The bell-shaped curve represents the opening of the virus and release of the genome as seen in the virus control in red. The virus treated with ferment of strain 8 or 14 are seen in pink and orange curves, respectively. The individual ferments do not contribute towards any background fluoresce when combined with SGII as seen with the indigo and green curves. This experiment was performed three times independently and this is a representative result. (**E**) Negatively stained TEM images of (i) CVA9 (1.6 × 10^10^ PFU/mL) and (ii) CVA9 treated with strain 14 ferment (10% v/v). Scale bar, 100 nm. The intact virus particles having bright center and empty/broken viral particles having darker center are highlighted with red and yellow color respectively in the control virus image.

Next, we set out to study in more detail what may be the mechanism of action. Time-of-addition-studies were carried out to understand whether the ferments only have a direct effect on virus particle or if they interfere also with cellular steps of infection. Three modes of infection were studied in the assay (Fig. 3A). For pre-infection, cells were first treated with the ferment for 1 h, then washed away and infected with the virus. In co-infection mode, virus and ferment mix was added directly on cells and incubated for 24 h. Finally, in the post-infection mode, cells were first infected with the virus for 1 h and then ferment was added. Based on the studies, it was evident that the ferments were not effective when added before or after virus infection on cells (Fig. 3B (i) and (iii)). However, when the ferment-virus mix was added on cells, the ferment showed antiviral activity (Fig. 3B (ii)). Based on these results, it was clear that the *Ganoderma* sp. ferment does not inhibit virus infection through acting on cellular targets in the used concentrations, nor does it interfere with the viral replication. Instead, the ferment seems to have a direct effect on the virus capsid.

To evaluate possible structural changes on the virions, a sucrose gradient assay was performed. A 5–20% sucrose gradient conveniently separates the virions into empty, intact, and intermediate/expanded particles as we have shown before^24,25^. To make the method very sensitive, we performed the assay using metabolically labelled virus (^35^SMet/Cys-CVA9), which was incubated with the *Ganoderma* sp. ferment (strain 14 or 17) for 1 h at 37 °C. Fresh virus control without the ferment and with no pre-incubation showed a single peak in the dense lower part of the gradient, demonstrating that the control virions were intact. If empty virions would appear in the preparation, those would be in the fractions around 8 to 10^25^. Another virus control which was incubated with the storage buffer for 1 h at 37 °C showed no major difference to the fresh virus control demonstrating that the 1 h incubation at 37 °C did not cause any structural changes to the virions. However, strikingly, when the virus was treated with the *Ganoderma* sp. ferments, we observed almost a complete loss of radioactivity (Fig. 3C). In the light of our recent results with polyphenols, this led us to think that the pre-treatment of the virus with the *Ganoderma* sp. ferment might be clustering the virus, leading to formation of virus aggregates, which led to strong sedimentation of the virions^26^. We observed a similar loss of radioactivity also in our earlier study upon formation of virus particle aggregates, when performing fractionation during a CsCl_2_ gradient^24^.

We then decided to study virus stability of enteroviruses in the presence of different strains of the ferment using Particle Stability Thermal Release Assay (PaSTry). In this assay, the slow stepwise heating of the virus in a PCR machine reveals the temperature at which the virus opens and releases its genome. A fluorescent RNA binding probe added to the reaction mix starts emitting fluorescence when in contact with the released genome of the virus. Our results showed that the control virus CVA9 gave a typical bell-shaped Melt curve (Fig. 3D). The melting temperature (Tm) was derived from the Melt peak (Supplementary Fig. S4) which was plotted using the derivative of relative fluorescence units (RFU) as a function of temperature (d(RFU)/dT). The melting temperature of CVA9 control virus was 51.5 °C, while the melting temperature of the CVA9 pre-treated with strain 8 or 14 (80% v/v) did not change much (52.5 °C and 52 °C; respectively), only the overall fluorescence strongly reduced as seen in Fig. 3D. These results suggested that the ferments strongly stabilized the viruses, and thus did not allow the viral genome to be released.

To confirm the structural stability of the enteroviruses upon treatment with ferments, we performed negative staining and subsequent TEM evaluation of the stained enterovirus (CVA9). The micrographs revealed that the control samples were mostly intact icosahedral viral particles with some occasional empty capsids (Fig. 3E (i)). Pretreatment of the viruses with strain 14 ferment caused the viruses to aggregate into small to medium size clusters (Fig. 3E (ii), Supplementary Fig. S6). On a closer look, the aggregates revealed intact particles and very few empty particles, just like the control samples. These results are in line with the outcome of the thermal assay: treatment with the ferment does not allow the virus to open and release its genome. Furthermore, the presence of aggregates explains the loss of radioactivity in the sucrose gradient from the fractions normally showing intact separated, non-clustered virions.

### Broadly acting antiviral and antibacterial potency of the ferments

As ferments potentially have broadly acting properties among viruses, we set out to test other viruses as well. The ferments of selected strains were first tested against the enveloped virus SARS-CoV-2, by treating the virus for 1 h at 34 °C with ferments before adding the virus on cells. Thereafter, real-time reverse transcriptase polymerase chain reaction (RT-qPCR) was executed for the RNA extracted from the ferment treated virus after 3 days of cultivation. qPCR measures then the presence of viral RNA in the cell supernatant in the form of Cq values and thus describes how well the virions have replicated in the cells. As Cq values are inversely proportional to the amount of viral RNA (cDNA) present in the sample, the lower the Cq values, the higher the amount of RNA present and vice versa. Our results showed that the virus control had a Cq value of 14.82, indicating a high amount of viral RNA after 3-days of infection. In contrast, the Cq values were considerably higher for the virus treated with strain 14 or 17 ferment indicating a significant reduction in the viral RNA (Table 2). Cq values were also used to calculate the logarithmic reduction of the infectivity (Table 2). Based on these calculations, we got a 4.6-to 5.8-log reduction in the viral RNA when virus was pre-treated with the strain 14 or 17 ferment. This result altogether provides confirmation of the high antiviral activity of strain 14 or 17 ferment against SARS-CoV-2.

**Table 2:** The effect of strain 14 (S14) and 17 (S17) ferments on SARS-CoV-2 infection in Vero-E6 cells.

Sample	Cq mean value	Difference in Cq value compared to VC	Log difference
S14 50% v/v	34.0307	19.2096	5.7808
S17 50% v/v	30.3808	15.5597	4.6794
VC	14.8211	-	-

Cq mean values of the test and virus control (VC) samples obtained from the qPCR are shown. These mean Cq values were used to calculate the difference between test and virus control samples, which was further used to calculate logarithmic RNA difference. VC, virus control; Cq, quantification cycle. The experiment was performed once with three technical repeats.

**Fig. 4:**
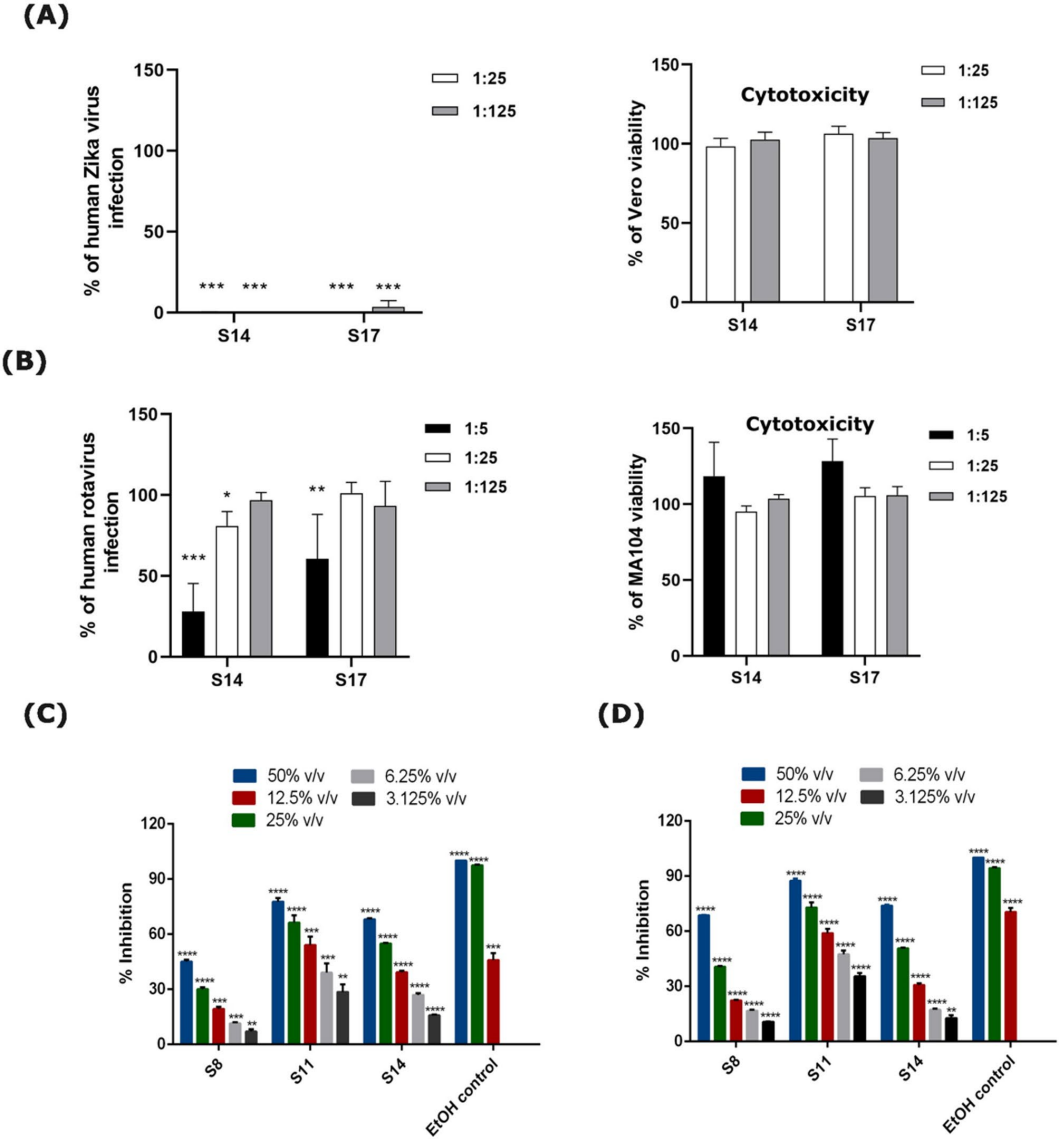
Broad-spectrum antiviral and antibacterial activity of the *Ganoderma* sp. ferment. Determination of antiviral activity of ferments of strain 14 or 17 against (**A**) ZIKV and (**B**) HRV, was performed by focus reduction assays on Vero cells or MA104 cells, respectively. Cells were seeded at a density of 13,000 cells/well in 96-well plates. The following day, mixtures of serially diluted *Ganoderma* sp. ferments (1:5, 1:25, or 1:125 parts) and virus at MOI of 0.02 focus-forming units/cell, were incubated for 1 h at 37 °C. These virus-*Ganoderma* sp. ferment mixtures were then added to cells for 1 h in the case of HRV or for 2 h in the case of ZIKV. Subsequently, this mixture was removed, fresh medium with diluted *Ganoderma* sp. ferment was added, and cells were incubated for 24 h at 37 °C. Infected cells were detected by indirect immunostaining. The percentage of infection was calculated by comparing treated and untreated wells. The results are expressed as mean and standard error of mean from two independent experiments. Statistical significance was calculated by performing one-way ANOVA, followed by Bonferroni test (*p < 0.05, **p < 0.01 and ***p < 0.001). (**C**) The antibacterial efficacies of the ferment of strain 8, 11 or 14 and the positive ethanol control against *E. coli* and (**D**) *S. aureus* strains after 50 min incubation. The results are expressed as mean and standard error of mean from three technical replicates in inhibition percentages and indicate the direct response of living bacteria to the samples. Statistical significance was calculated by performing one-way ANOVA, followed by Bonferroni test (*p < 0.05, **p < 0.01 and ***p < 0.001).

Next, the ferments were tested against the enveloped Zika virus (ZIKV) and non-enveloped Human rotavirus (HRV) infection by incubating the virus-ferment mix for 1 h at 37 °C. The ferments of strain 14 or 17 completely inhibited the ZIKV infection even at the lower tested concentration (Fig. 4A). In case of ferment treated HRV, the antiviral efficacy increased with higher amount of ferment in the assay (Fig. 4B). These studies confirm the broad-spectrum antiviral activity of *Ganoderma* sp. ferments.

The antibacterial efficacies of the ferment of strain 8, 11 and 14 against bacterial biosensor *S.aureus* and *E.coli*, which represent the leading pathogens of healthcare-associated infections and bacteremia^27^ are shown in Fig. 4C and **D**. All the fungal cultivation samples showed high activities against both strains. Maximum inhibitions against *S. aureus* were 68.54%, 87.37%, and 73.85% for strain 8, 11 and 14, respectively. Whereas the inhibition maximum values against *E. coli* were 45.02%, 77.57% and 68.02% for strain 8, 11 and 14, respectively. These are in the same range as the ethanol control of 12.5% (v/v), which gave inhibition of 70.40% with *S. aureus* and 45.78% for *E. coli*. Couple of conclusions can be drawn; strain 11 was the most efficient strain against both gram-negative *E. coli* and gram-positive *S. aureus* whereas strain 8 seemed to possess the lowest activity against both bacterial strains used. Test results also show that the gram-positive *Staphylococcus aureus* is more susceptible to the fungal ferments.

### Potential bioactive components of the ferments

Terpenoids have been recognized as bioactive components in *Ganoderma* sp^13^. We also suspected terpenoid compounds to be responsible for the antimicrobial efficacy of the ferments. So, we set out to analyze selected ferments 8, 11, 14 and 17 for the presence of terpenoids using ultrahigh-performance liquid chromatography ultrahigh-resolution tandem mass spectrometry (UHPLC-HR-MS/MS). In total, 72 metabolites were tentatively identified, of which majority belonged to triterpenoids (Supplementary Table S1). All the components were detected in the negative ion mass spectra as deprotonated molecular ions ([M‒H]^‒^). In positive ionization, over a half of the components were found either as protonated molecular ions, sodium adducts or protonated molecular ions with one to three water cleavages ([M + H]^+^, [M + Na]^+^ and/or [M-nH_2_O + H]^+^, *n* = 1‒3, respectively). In addition to the selected ferments, culture medium and authentic triterpenoid standards platycodigenin, cucurbitacin IIb and ganoderic acid G, previously reported components of *Ganoderma* sp. ferment or fungus^28,29^were screened with the UHPLC-MS/MS. Based on the retention times, none of these reference compounds analyzed were present in the ferments. Culture medium was found to contain soyasaponin I (**67**) and traces of soyasaponin type compounds **38**, **51**, and **65**. Our hypothesis is that these soyasaponins originate from the soya peptone used in the preparation of the liquid culture medium. However, ferments contained also other soyasaponin type compounds, **18**, **19** and **21**, apart from the ones found from the culture medium. These components are most likely metabolites from the fermentation process with the *Ganoderma* sp. strains.

The metabolite content in each ferment is presented as heatmap in Supplementary Fig. S2. The content of individual compounds varied between ferments, but all 72 compounds were present in every ferment studied, even though huge variations were detected in their contents. The main components in the ferments were compounds **55** and **56**, i.e. isomers of cucurbitacin IIb. Further, compounds **70** and **71** showed high contents in ferment of strain 17 but were present to a lesser extent in other ferments, especially ferment of strain 8.

## Discussion

We show here that the ferments from the selected *Ganoderma* sp. (Basidiomycota) strains belonging to the *G. lucidum* complex and originating from Finland contain excellent antiviral properties against both non-enveloped and enveloped viruses. The results thus demonstrate potent broadly acting efficacy against viruses tested and give a great promise for their future use. The fact that the ferments could combat against a high load of the very stable non-enveloped enteroviruses at already less than 1 min contact time, demonstrates the exceptionally high antiviral efficacy of the ferments. Additionally, the tested fungal ferments were also active against the gram-negative and gram-positive bacteria, demonstrating their broadly acting antimicrobial efficacy.

The antiviral efficacy was fungal strain-dependent even though submerged fermentation process conditions were identical for all the *Ganoderma* sp. strains tested. Only the most promising candidates from larger screenings were chosen for this study. In addition to validating the bioactive strain hits with other enterovirus strains, this study further revealed the mechanisms of action towards non-enveloped viruses. The results clearly showed that with the used concentrations, the ferments acted mainly through direct effect on enterovirus capsid. This was proven by several lines of evidence: time-of-addition-assays, thermal assay, radioactive sucrose gradient assay and negative staining and TEM. The ferments caused aggregation of the virions which likely also led to a lower amount of entry to the cells as well as contributed to higher stability, which prevented the virions from releasing the genome and thus replication.

The Nancy strain of CVB3 has a collapsed pocket while CVB1 and CVA9 have a wider pocket^23^. The lower efficacy of the ferments towards CVB3 Nancy strain in comparison to CVA9 and CVB1 strongly suggest that the typical docking site of direct capsid binders on enteroviruses in general, the hydrophobic pocket, is also here the most likely major binding site of the bioactive components of the ferments. While the hydrophobic pocket may not be the only docking site, it usually shows strong binding of various capsid binders leading to strong stabilization of the enteroviruses^23,26^. The somewhat lower activity of the ferments against the tested rotavirus suggests that the bioactive components binding directly to the rotavirus capsid are less in number and/or less strong in binding to the capsid.

The ferment is a mixture of several potentially bioactive components. Also, fungal-derived terpenoids have been identified as one of the largest groups of bioactive natural compounds^30^. This prompted us to make a metabolic evaluation of the high-efficacy and lower efficacy ferments, which showed several potential components that could on their own or together lead to excellent antiviral activity. Since the metabolic profiling suggested differences in the metabolite content, but not in composition between the low and high efficacy ferments, this suggests to us that the content of some selected metabolites could be behind the high antiviral efficacy. However, it is still to be shown in future studies whether some individual compound or the combination of several compounds exudated to the medium during fermentation are responsible for the antiviral efficacy of the ferments. Over 430 secondary metabolites have been identified in fungal species belonging to the *Ganoderma* genus^21^ from which triterpenoids have shown antiviral activities against HIV-1^31,32^. The antiviral activity of *Ganoderma lucidum* triterpenoids has been evaluated also against EV71 and it was suggested to block the virus uncoating^16^. *Ganoderma lingzhi* has also been reported to show antiviral activity by inhibiting neuraminidase activity of H5N1 and H1N1^33^. In addition, fungal polysaccharides have been reported to have antiviral activities^30^. In general, basidiomycetous fungal strains are not genetically tractable and yet very little is known about their terpenoid biosynthetic pathways, although they are known to produce a huge diversity of terpenoids^22,34^.

Our results show that ferments of selected strains of *Ganoderma* sp. belonging to the *Ganoderma lucidum* complex and originating from Finnish forests are a promising source of antiviral and antibacterial compounds that require submerged fermentation technology for their production. Not all the strains had the same performance against viruses, suggesting that the results cannot be generalized to a fungal species level. Furthermore, differences in the metabolic components and their efficacy are likely the key to their differences and those aspects will be studied in future studies in more detail. *Ganoderma* sp. ferments showed broadly acting antiviral activity and a strong, direct effect on the enterovirus virions, causing virus clustering and stabilization leading to prevention of virus entry and genome release. Future prospects include investigation about the mechanism of action of ferments against bacteria and fractionation of ferments in order to obtain isolated compounds. However, it is highly possible that the observed activities can be caused by synergistic effects of several compounds within the ferments. synergistic effects for forest-derived bioactive extracts were proposed as a reason for example by Willför et al. (2003), who found that the antioxidant potency of conifer knotwood extracts was higher than that of their predominant pure compounds^35^. Additionally, triterpenoids have been indicated to have synergistic effect against bacteria with other active compounds, such as flavonoids and antibiotics^36,37^. The results thus show great promise for the future use of *Ganoderma* sp. ferments in combating virus and also bacterial infection for several applications.

## Materials and methods

### Fungal strains used in this study

Polypore basidiocarps belonging to the *Ganoderma* genus growing on *Betula pubescens* and *Picea abies* wood stumps were collected in Satakunta and Uusimaa provinces in Finland. Dikaryotic strains were isolated from the basidiocarps to pure cultures and identified to the species complex level based on fungal DNA barcode (rDNA ITS) region. The species identity of the strains has been studied and discussed in our recent multigene phylogenetic and morphological characteristics study^38^. According to the phylogenetic research, all the strains used in this study belong to the *Ganoderma lucidum* complex, but the accurate species recognition is not resolved; therefore, the strains are referred as *Ganoderma* sp. In total of 17 strains of *Ganoderma* sp. were screened in this study. All the living strains are maintained at the Natural Resources Institute Finland (Luke) culture collection in Helsinki, Finland.

### Preparation of submerged *Ganoderma* sp. cultures

The isolates were subcultured and maintained on modified orange serum agar (MOS-agar)^39^ in 9 cm Petri dishes at 22 °C in dark for two weeks prior to liquid culturing. Cultures (approximately 2 cm^2^ mycelium cut from the actively growing colonies on MOS) were grown in 100 mL liquid culture medium, which consisted of the following components (g/L): glucose 35, soya peptone 5, yeast extract 2.5, KH_2_PO_4_ 1, MgSO_4_ × 7H_2_O 0.5, vitamin B1 0.05, pH adjusted to 5.5 (optimal medium for *Ganoderma* species)^40^. Cultures were grown in two-stage cultivation process, combining shake-flask fermentation with static culture^19^. In the first stage, the cultures were shaken at 125 rpm at 25 °C for 7 days. This was followed by seven weeks fermentation in static culture at 25 °C in the dark. The cultivations were done as five biological replicates. After the fermentation the liquid was harvested from the mycelium and sterile filtered through 0.2 μm filters. The filtered liquid is referred as “ferment” from now on in this paper.

### Reference compounds

The triterpenoid compounds cucurbitacin IIb (CAS 50298-90-3), platycodigenin (CAS 22327-82-8), and ganoderic acid G (CAS 98665-22-6) were all acquired from Chem-Faces, Wuhan, China.

### Ferment analysis by UHPLC-HR-MS/MS

The metabolite profiling of the ferment of strains 8, 11, 14 and 17 was performed by UHPLC-HR-MS/MS according to a previously published method with minor modifications^41^. Briefly, the LC-MS system consisted of an Acquity UPLC (Waters Corp.) connected to a quadrupole-Orbitrap mass spectrometer (QExactive™, Thermo Fisher Scientific GmbH) via heated electrospray ionization (HESI) source. The separation of ferments’ components was achieved with an Acquity UPLC BEH Phenyl column (2.1 × 100 mm, 1.7 μm, Waters Corp.). The binary mobile phase consisted of acetonitrile (A) and 0.1% (v/v) formic acid in water (B). The elution profile was: 0–0.5 min, 0.1% A in B (isocratic); 0.5–5 min, 0.1‒30% A in B (linear gradient); 5–7 min, 30‒40% A in B (linear gradient); 7–7.1 min, 40‒90% A in B (linear gradient); 7.1–9.1 min, 90% A in B (isocratic). Flow rate was 0.5 mL/min and injection volume 5 µL. Ferments were stored in 5 °C and filtered through 0.2 μm PTFE filters prior analysis.

The MS was operated in both positive and negative ion modes and the spray voltage was set to 3.8 kV or ‒3.0 kV, respectively. Capillary temperature was set to 380 °C. Mass range reached from *m/z* 150 to *m/z* 2250. The in-source collision-induced dissociation (CID) was set 0 eV to avoid excessive fragmentation of the ferment components and facilitate the detection of molecular ions. Stepped normalized collision energies 20, 50 and 80 eV were applied for MS/MS analyses. Thermo Xcalibur Qual Browser software (version 4.1.31.9) was utilized for data processing. Data was further processed with Compound Discoverer 3.1 (Thermo Fisher Scientific Inc.) and MZmine (version 2.53) software for tentative identification of the ferments’ components. The confidence level of identified metabolites was expressed as Metabolomics Standards Initiative (MSI) value initially proposed by Sumner et al.^42^. Triterpenoid standards, platycodigenin and cucurbitacin IIb, previously identified from *Ganoderma tsugae* ferment^28^ and ganoderic acid G, previously characterized from *Ganoderma lucidum* (FR.) KARST^29^were used as triterpenoid reference compounds in the analyses and CID of 30 eV was used for these known compounds.

### Cells

Human alveolar basal epithelial adenocarcinoma (A549) cells and Vero E6 were obtained from American type culture collection (ATCC, Manassas, VA, USA). The A549 cells were propagated in Dulbecco’s Modified Eagle Medium (DMEM) (Gibco, Paisley, UK) whereas Vero E6 cell lines were grown in Eagle’s Minimum Essential Medium (MEM) (Gibco, Paisley, UK). Both MEM and DMEM were supplemented with 10% heat-inactivated Fetal Bovine Serum (FBS, Gibco, Paisley, UK), 1% L-GlutaMAX (Gibco, Paisley, UK) and 1% antibiotics (penicillin/streptomycin) (Gibco, Paisley, UK) and the cells were cultured in a humidified 5% CO_2_ incubator at 37 °C. African green monkey kidney epithelial cells MA104 (ATCC^®^ CRL-2378.1) and Vero (ATCC CCL-81) were propagated in DMEM (Gibco-BRL, Gaithersburg, MD) supplemented with 10% FBS.

### Viruses

Coxsackievirus B3 (CVB3; Nancy strain), Coxsackievirus B1 (CVB1, CONN 5 strain) and Coxsackievirus A9 strain (CVA9; Griggs strain), were obtained from ATCC. They were produced and purified as described before^24,43^ with one exception of adding 0.1% (v/v) TWEEN^®^ 80 (Sigma-Aldrich, Steinheim, Germany) during the freeze–thaw cycle. SARS-CoV-2 (SARS-CoV-2/Finland/1/2020) used was isolated from the first Covid-19 patient in Finland^44^. Human rotavirus (HRV) strain Wa (ATCC^®^ VR-2018) was activated with 5 µg/mL of porcine pancreatic trypsin type IX (Sigma, St. Louis, Mo) for 30 min at 37 °C and propagated in MA104 cells by using DMEM containing 0.5 µg of trypsin per mL as previously described^45^. Zika virus (ZIKV) (1947 Uganda MR766) was generated by transfection of 293T cells with the plasmid pCDNA6.2 Zika MR766 Intron3115 HDVr MEG 070916 5, as previously described^46^. The virus was then propagated in Vero cells and titrated by plaque assay.

### Antiviral activity assay for enteroviruses

The screening of the ferments to determine their antiviral activity against enteroviruses was performed using the cytopathic effect inhibition (CPE) assay as described before^26^. Briefly, A549 cells at a density of 20,000 cells/well were cultured in 100 µL of DMEM supplemented with 10% FBS, 1% GlutaMAX and 1% penicillin/streptomycin antibiotics on a 96-well flat-bottomed microtiter plate (Sarstedt, Numbrecht, Germany) for 24 h in 5% CO_2_ and 37 °C. Next day, 10% v/v of the virus was pre-treated with the 90% v/v of the ferment for 1 h at 37 °C (CVB3, CVB1 and CVA9 titer in the virus-*Ganoderma* sp. ferment mix was 7 × 10^6^ PFU/mL, 5 × 10^6^ PFU/mL, and 3 × 10^8^ PFU/mL, respectively). The virus-*Ganoderma* sp. ferment mix was further diluted using DMEM by a factor of ten to get a final multiplicity of infection (MOI) of 2 for CVB3 and CVB1 and 106 for CVA9. Following this, the mix was added on the cells and incubated for another 24 h at 37 °C. Mock infection (without virus and the ferment) and virus control (without ferment) were used as negative and positive controls for the experiment, respectively. The cytopathic effect of the virus was monitored using the light microscopy. Next day, after two washes with PBS, cells were fixed and stained for 10 min using CPE dye (0.03% crystal violet, 2% ethanol and 3% formaldehyde in water). The stained viable cells were then washed twice with water, following which they were lysed using a lysis buffer (0.8979 g of sodium citrate and 1 N HCl in 47.5% ethanol). Finally, the absorbance of the viable cells in the 96-well plate was measured spectrophotometrically at 570 nm using the PerkinElmer VICTOR™ X4 multilabel reader (PerkinElmer, Turku, Finland).

### Endpoint Dilution assay

A549 cells were seeded at a density of 10,000 cells/well on the 96-well flat-bottomed microtiter plate and incubated for 24 h in 5% CO_2_ and 37 °C. Next day, CVA9 (1:250 dilution) was mixed with the ferment of strain 14 (10% v/v) and incubated for 1 h at 37 °C. A virus control with similar amount of virus without the ferment was also incubated. After the incubation, the virus-*Ganoderma* sp. ferment solution was serially diluted by ten-folds (ten dilutions in total). Each of the dilution was added in replicates of eight on the cells and incubated for 3 days at 37 °C. Following the incubation, the cells were stained with the crystal violet dye (0.13% crystal violet, 5% ethanol and 5% formaldehyde in PBS) for 10 min to differentiate between the healthy and infected cells. Finally, the virus titers were calculated using the Reed-Muench method^47^ and expressed as particle forming units (PFU) per mL.

### Antiviral activity assay for SARS-CoV-2

Vero-E6 cells were cultured in a 100 µL of MEM supplemented with 10% FBS, 1% GlutaMAX and 1% penicillin/streptomycin antibiotics at a density of 50,000 cells/well on a 96-well flat-bottomed microtiter plate for 24 h at 37 °C. Following day, SARS-CoV-2 was pre-treated with ferment of strain 14 or 17 (50% v/v) and incubated for 1 h at 34 °C. The virus titer in the virus- *Ganoderma* sp. ferment mix was 200 PFU/mL. Handling of the virus was carried out at the BSL-3 facility at the University of Helsinki, Finland. After the incubation, the virus- *Ganoderma* sp. ferment mix was further diluted using MEM by a factor of ten to get a final MOI of 0.00002 and then added on the cells for 2 h at 34 °C. Following the incubation, the cells were aspirated, fresh media was added and incubated for another 3 days at 34 °C. Finally, the supernatant from the cells was collected and transferred to a new 96-microtiter plate for viral RNA extraction. The extraction was done using a chemagic Viral RNA/DNA kit (PerkinElmer). After the RNA extraction, we performed RT-qPCR to qualitatively detect viral nucleic acid. This was performed using SARS-CoV-2 RT-qPCR reagent kit (PerkinElmer). From the SARS-CoV-2 qPCR experiment, Quantification cycle (Cq) values were used to calculate the RNA difference between the test samples and reference virus control using the equation shown below. Then the RNA difference was calculated and presented in its logarithmic form.

$$RNA{\text{ }}difference\,=\,0.{\text{9646}}{{\text{e}}^{0.{\text{6948}}x}}$$

where *x* is the difference in the Cq values between the mean of ferment treated and untreated samples.

### Antiviral activity assay for human rotavirus (HRV) and Zika virus (ZIKV)

Anti-HRV and anti-ZIKV activity of ferment of strain 14 or 17 was determined by focus reduction assays on MA104 cells or Vero cells, respectively. Cells were seeded at a density of 13,000 cells/well in 96-well plates. The following day, mixtures of serially diluted *Ganoderma* sp. ferments (1:5, 1:25, or 1:125 parts) and virus at MOI of 0.02 focus-forming units/cell, were incubated for 1 h at 37°C. These virus-*Ganoderma* sp. ferment mixtures were then added to cells for 1 h in the case of HRV or for 2 h in the case of ZIKV. Subsequently, this mixture was removed, fresh medium with diluted *Ganoderma* sp. ferment was added, and cells were incubated for 24 h at 37°C. After this time, necessary for viral replication, cells were fixed with cold acetone-methanol (50:50). HRV- and ZIKV-infected cells were detected by indirect immunostaining using specific primary antibodies, and the secondary antibody peroxidase-conjugated AffiniPure F(ab’)2 Fragment Goat Anti-Mouse IgG (H + L) (Jackson ImmunoResearch Laboratories Inc., Baltimore Pike, West Grove, PA, USA). The number of infected cells was counted, and percentages of infection were calculated by comparing ferment treated and untreated wells. This assay was performed twice or thrice independently.

### Time of addition studies

Time of addition studies were performed using the CPE inhibition assay as described above. For this assay, three modes of infection were designed and studied. In the pre-infection mode, cells were incubated with the ferment of strain 14 (10% v/v) for 1 h at 37 °C. After the incubation, cells were washed briefly and then infected with CVA9 (MOI − 160) and incubated for 24 h at 37 °C. In the co-infection mode, a mix of virus (3 × 10^8^ PFU/mL) and strain 14 ferment (90% v/v) was prepared and then diluted 10 times using the media before adding it on the cells (MOI-160) and incubated for 24 h at 37 °C. In the post-infection mode, cells were infected with CVA9 (MOI − 160) for 1 h at 37 °C. After the infection, excess virus was removed by repeated washing. Then, media having strain 14 ferment (10% v/v) was added and incubated for 24 h at 37 °C. Virus control and mock infection were used as controls during each of the different modes of infection studied. This experiment was performed twice independently.

### Metabolic labeling of CVA9

The metabolically labeled virus was produced as described before^26^. Briefly, confluent GMK cells propagated in 75 cm^2^ bottles were infected with CVA9 diluted in low-methionine-cysteine medium (MP Biomedicals, Illkrich, France) supplemented with 1% FBS and 1% Glutamax. Following 3 h of virus infection, 40 µCi/mL of radioactive^34^S] methionine/cysteine (PerkinElmer, Boston, MA, USA) diluted in low-methionine-cysteine medium supplemented with 1% FBS was added to the cells. The virus infection was let to proceed for 24 h at 37 °C, followed by three freeze–thaw cycles and then centrifuged at 2500 × *g* for 10 min to obtain a pellet. TWEEN^®^ 80 (0.1% v/v) was added to the pellet and incubated on ice for 30 min, followed by centrifugation for 10 min with 4000 × *g* at 4 °C. The supernatant obtained was added on a 2 mL sucrose cushion (40%) and ultracentrifuged at 151,263 × *g* for 2.5 h (4 °C) using a SW-41 rotor. All the liquid up until the cushion and first 500 µL fraction from the top were discarded. The next 3 × 500 µL fractions were collected and pelleted using a 70Ti rotor at 90,140 × *g* for 2 h (4 °C) to concentrate the virus. This was followed by layering the concentrated virus on top of the 10 mL sucrose gradient (5–20%) and ultracentrifuged with a SW-41 rotor at 151,263 × *g* for 2 h (4 °C). Fractions (500 µL) were collected from the top and a small aliquot from each fraction was mixed with a scintillation cocktail UltimaGold™ (PerkinElmer, Waltham, MA, USA) for measuring the radioactivity (counts per minute) using a Tri-Carb^®^ 2910TR liquid scintillation analyzer (PerkinElmer, Downers Grove, IL, USA). The 160 S-containing fractions were pooled and used for the experiments (7000 CPM/µL: 1.4 × 10^9^ PFU/mL).

### Gradient assay

Metabolically labeled CVA9 (70,000 CPM corresponding to 1.4 × 10^8^ PFU/mL) was pre-treated with ferment of strain 14 or 17 (90% v/v) in a total volume of 100 µL for 1 h at 37 °C. A virus control (without the ferment but the same treatment) and fresh virus (without the ferment and without treatment) were used as positive controls for the assay. The test and control samples were further diluted to reach a final volume of 1 mL using PBS-MgCl_2_ buffer (PBS containing 2 mM MgCl_2_). The samples were then layered over 10 mL sucrose (5–20%) gradients and ultracentrifuged at 151,263 × *g* (average g value) for 2 h (4 °C) using the SW-41 rotor. Finally, gradients were fractionated from top to bottom (500 µL fractions) and mixed with a scintillation cocktail for measuring the radioactivity (counts per minute) using a PerkinElmer Tri-Carb^®^ 2910TR liquid scintillation analyzer.

### Particle stability thermal release assay (PaSTRy)

The stability of the enteroviruses was studied using the PaSTRy assay. The assay was done as described before^48^. A reaction mixture of 50 µL containing 1 µg of CVA9 and strain 8 or 14 ferment (80% v/v) was made in PBS and incubated for 1 h at 37 °C. Post incubation, 10X SGII (Invitrogen) diluted in ddH_2_O was added to the reaction mix, and the entire mixture was aliquoted into the thin-walled PCR plate (Agilent, Amstelveen, Netherlands). The thermal cycler (BioRad C100, Helsinki, Finland) recorded the fluorescence in quadruple from 20 to 90 °C with 0.5 °C intervals. The fluorescence data output was extracted from the BioRad CFX manager (2.1 software, accessed on 22.3.2022) and the graph was plotted in GraphPad PRISM. The melt curve was obtained by plotting the relative fluorescence units (RFU) versus temperature (T).

### Immunofluorescence labeling and microscopy

A549 cells were seeded at a density of 8000 cells/well on the 96-well flat-bottomed microtiter plate (Fischer Scientific, Finland) and incubated for 24 h in 5% CO_2_ and 37°C. Next day, the virus (CVA9–1.2 × 10^8^ PFU/mL) was pre-treated with ferment of strain 8 or 14 (10% v/v) by preparing a virus-ferment mix in a buffer (PBS having 2 mM MgCl_2_) and incubated for 1 h at 37°C. After the incubation, the mix was diluted by a factor of 10 using DMEM, then added on A549 cells (MOI of 100) and incubated for 6 h at 37°C, before fixing with 4% paraformaldehyde for 30 min. The cells were then permeabilized with 0.2% Triton X-100. Following this, they were treated with primary antibodies: rabbit antibody labeling the CVA9 capsid protein (kind gift from Dr. Merja Roivainen) and mouse J2 against dsRNA of the virus (Scicons). After 1 h of incubation, cells were washed with PBS to remove excess primary antibody and then treated for 30 min with secondary antibodies: goat anti-rabbit Alexa 555 (Invitrogen) or goat anti-mouse Alexa 488 (Invitrogen, Life Technologies, USA). Secondary antibodies were washed with PBS and cell nuclei were stained with 4’,6-diamidino-2-phenylindole (DAPI, Molecular Probes, Life Technologies, USA) in PBS. Samples were imaged with Nikon A1R confocal microscope. The imaging was carried out with the 40× objective (NA 1.25), and 405 nm diode laser, 488 nm multiline argon laser and 561 nm sapphire laser. Laser power and detector amplification settings were optimized for each channel. Virus protein and dsRNA channels were adjusted according to the cell control to exclude antibody background. Images were visualized using the software Fiji2 (ImageJ). CellProfiler 4.2.1 was used to determine the number of infected cells in a sample. First, the nuclei were identified as primary objects using the Otsu thresholding method. Next, the infected cells were identified as secondary objects using the previously identified nuclei as a reference. Manual thresholding method was used to differentiate the background from the virus protein signal. Finally, the area and intensity of the secondary objects were measured, and data exported to excel where a threshold was set manually to differentiate infected from non-infected cells. Quantification was done to calculate the infection (%) by comparing the infected cells of virus control with that of test samples. At least 500 cells per sample were analyzed in the experiment.

### Transmission Electron microscopy

A reaction mixture of CVA9 (1.6 × 10^10^ PFU/mL) and strain 14 ferment (10% v/v) in PBS was incubated for 1 h at 37 °C. Untreated virus of the same amount was used as a virus control. During incubation, formvar coated copper grids were glow discharged using the EMS/SC7620 Mini sputter coater (Electron Microscopy Sciences, Hatfield, PA, USA). Post incubation, the samples were coated on the grids for 30 s, after which the excess was blotted away using a Whatman paper (Whatman 3 MM). The grids were negatively stained with 1% (w/v) phospotungstic acid (PTA) for 10 sec. The samples were imaged with the JEOL JEM-1400 transmission electron microscope (TEM) (JEOL, Tokyo, Japan) equipped with a field emission gun, LaB_6_ filament, operating at a voltage of 80 kV, in the BF- TEM imaging mode.

### Antibacterial assay

The antibacterial activity of terpenoids and *Ganoderma* sp. ferment were examined using recombinant bacterial biosensors *Staphylococcus aureus* RN4220 + pAT19 (gram-positive) and *Escherichia coli* K12 + pcGLS11 (gram-negative) strains, first described by Vesterlund and colleagues^49^. Both strains have been genetically modified to produce a constant luminescent light signal as a part of their normal metabolism and the presence of antibacterial substances can be detected as a decrease in the luminescent light production, which can be monitored in real-time. The ferment of strain 8, 11 and 14 were tested in 50, 25, 12.5, 6.25, and 3.13% (v/v) per microplate well and diluted with double distilled water. The methodology has been previously reported^50^. In brief, bacteria were stored in -80 °C and, to obtain bacterial stocks, cultivated for approximately 16 h at 30 °C and 37 °C on lysogeny agar (LA) plates (tryptone 10 g/L; yeast extract 5 g/L; NaCl 10 g/L; and agar 15 g/L) supplemented with 10% sterile phosphate buffer (PB) (1 M, pH 7.0) and 100 µg/mL ampicillin (*E. coli*) or 5 µg/mL erythromycin (*S. aureus*). A single colony of strains was cultivated in lysogeny broth (LB) medium with same supplementations as in LA plates for approximately 16 h at 30 °C (*E. coli*) and 37 °C (*S. aureus*) to obtain approximate optical densities OD_600_ = 1.0 (corresponding to ca. 8 × 10^8^ CFU/mL) and OD_600_ = 0.2 (corresponding to ca. 3 × 10^8^ CFU/mL), respectively. Double distilled water (negative control), ethanol (in three concentrations 50%, 25%, and 12.5% (v/v) per microplate well, positive control), and ferment samples (in five concentrations) were all pipetted in 50 µL aliquot triplicates into opaque white polystyrene microplates. The same volume of bacterial cultivation was added into same wells and the luminescence production was then monitored using a Varioskan Flash Multilabel device (Thermo Scientific) once every 5 min for a total of 60 min at room temperature and the plate was briefly shaken before every measurement. The results are obtained in relative light units (RLU) averages and drawn as inhibition% (inhibition% = (RLU_NegativecControl_ – RLU_sample_)/ RLU_NegativeControl_ × 100%)^51^ at a time point of 50 min of measurement.

### Statistical analysis

Statistical analysis was performed using GraphPad Prism 6 (GraphPad Software, San Diego, CA, USA). Data are presented as mean + standard error. Statistical significance was calculated by performing one-way ANOVA followed by the Bonferroni test (* *p* < 0.05, ** *p* < 0.01, *** *p* < 0.001 and **** *p* < 0.0001).

## Conclusion

Ferments of *Ganoderma* sp. show broad-spectrum antimicrobial activity against non-enveloped and enveloped viruses as well as against gram-positive and gram-negative bacteria. Enteroviruses were inhibited within one minute of pre-treatment with the ferments at room temperature. The ferments showed strong inhibition by directly acting on the virus surface and causing clustering of the enterovirus particles. This led to stabilization of the virus capsid and prevented its genome release. Chromatographic separation and metabolic profiling of the ferments confirmed the presence of several terpenoids which are known to contain excellent bioactivities. *Ganoderma* sp. ferments serve as a safe and sustainable option for several antimicrobial applications for the future.

## Electronic supplementary material

Below is the link to the electronic supplementary material.

Supplementary Material 1

## Data Availability

The data is available for the public upon request from the corresponding author.
