# Supplementary material for: Selected strains of the Ganoderma lucidum complex from Finnish forests have excellent broadly acting antiviral properties
Source: Sci Rep. 2025 Jul 2;15:23565. doi: 10.1038/s41598-025-08377-5 (PMC12223054; doi:10.1038/s41598-025-08377-5)
Supplement: Supplementary file 1 — Supplementary Material 1 [file 41598_2025_8377_MOESM1_ESM.pdf]

Supplementary material

Selected strains of the *Ganoderma lucidum* complex from Finnish forests have excellent broadly acting antiviral properties

Dhanik Reshamwala[1], Sailee Shroff[1], Jaana Liimatainen[2], Jenni Tienaho[2], Ilari Kuukkanen[3], Mira Laajala[1], Andrea Civra[4], Rachele Francese[4], Pyry Veteli[2], Marta Cortina-Escribano[5], Tytti Sarjala[2], Maarit Karonen[3], David Lembo[4], Riikka Linnakoski[2] ††, Varpu Marjomäki[1] ††*

[1] Department of Biological and Environmental Science, Nanoscience Center, University of Jyväskylä, Jyväskylä FI-40014, Finland
[2] Natural Resources Institute Finland (Luke), Helsinki FI-00790, Finland
[3] Natural Chemistry Research Group, Department of Chemistry, University of Turku, Turku FI-20014, Finland
[4] Department of Clinical and Biological Sciences, University of Turin, Turin I-10043, Italy
[5] Natural Resources Institute Finland (Luke), Joensuu FI-80130, Finland
††shared last authors

* Corresponding author
Varpu Marjomäki, Professor in Cell and Molecular Biology, Vice-head
University of Jyväskylä,
Department of Biological and Environmental Science/Nanoscience center
Survontie 9C,
FI-40500 Jyväskylä, Finland
+358-40 5634422
varpu.s.marjomaki@jyu.fi
https://www.jyu.fi/vmarjomaki

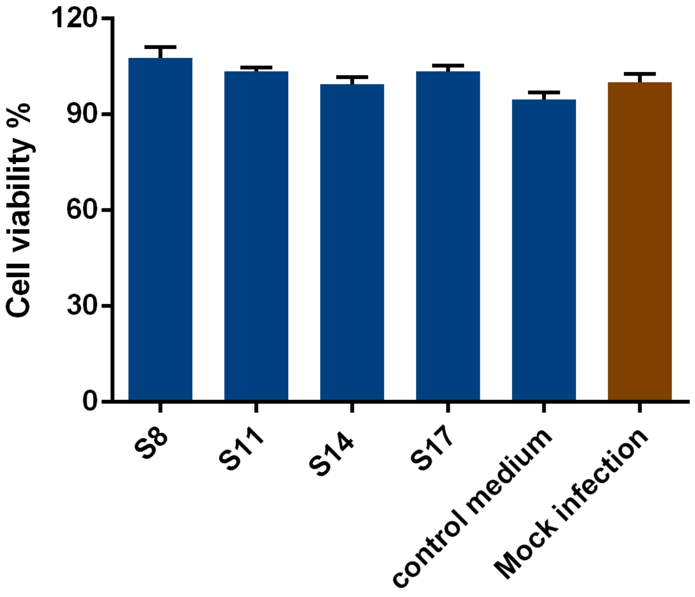

Supplementary Fig. S1 Cytotoxicity measurement of the selected hit ferments. The ferments were incubated in A549 cells (90 vol/vol) for 24 h at 37 °C and thereafter analyzed by cytopathic effect (CPE) inhibition assay. The test samples are normalized against the mock infection. Results shown are expressed as average values of three technical repeats + standard error of mean. Control medium here is the cultivation medium where ferments have been produced for several weeks.

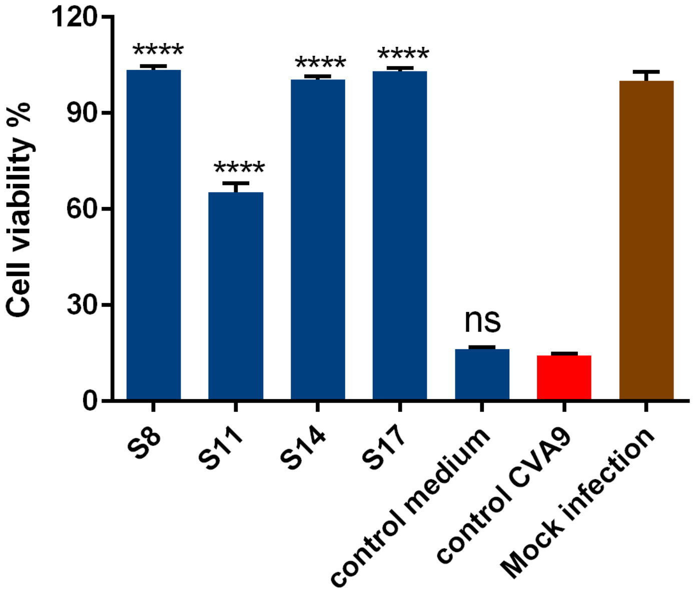

Supplementary Fig. S2. Effect of the growing medium (control medium) on the antiviral efficacy. The hit ferments and the medium used for fermenting the samples were incubated with CVA9 (90 % of the ferment and 10% of the virus solution) for 1 h at 37 °C and thereafter added in A549 cells for further 24 h and analyzed by cytopathic effect (CPE) inhibition assay. The results shown are expressed as average values of three technical repeats + standard error of mean. Statistical significance of the test samples against the virus control was calculated by performing one-way ANOVA, followed by Bonferroni test (*p < 0.05, ***p < 0.001 and ****p < 0.0001).

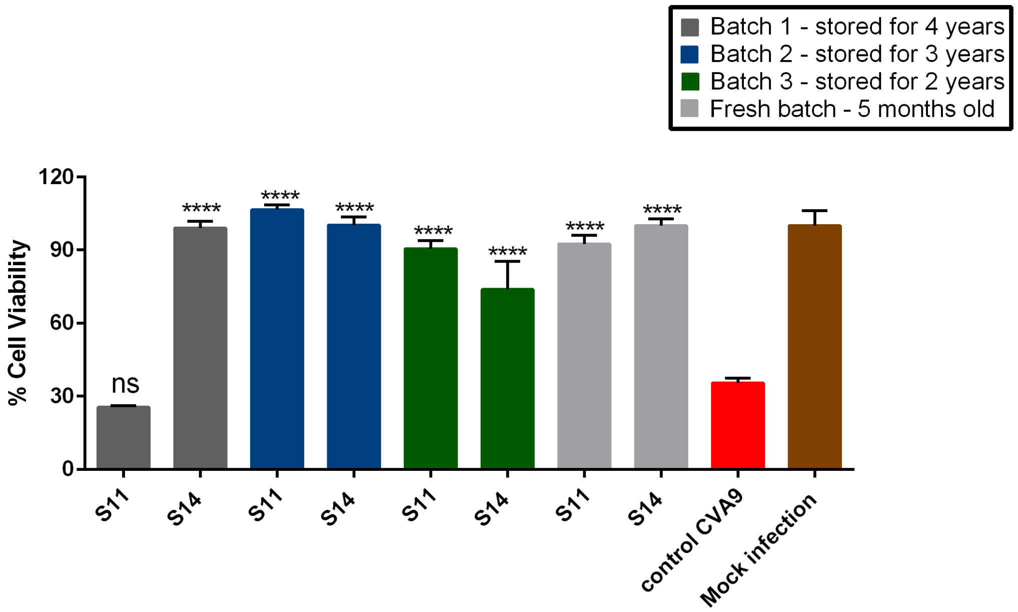

Supplementary Fig. S3. Stability of the antiviral efficacy due to storage of the ferments. Selected hit ferments (S11, S14) have been stored for 5 months, 2 years, and 3 years at +4°C and for 4 years at -20°C. The ferments were then tested for antiviral stability by incubating with CVA9 (90 % of the ferment and 10% of the virus solution) for 1 h at 37°C and thereafter adding in A549 cells for further 24 h and analyzing by cytopathic effect (CPE) inhibition assay. The results shown are expressed as average values of three technical repeats + standard error of mean. Statistical significance of the test samples against the virus control was calculated by performing one-way ANOVA, followed by Bonferroni test (*$p < 0.05$, ***$p < 0.001$ and ****$p < 0.0001$).

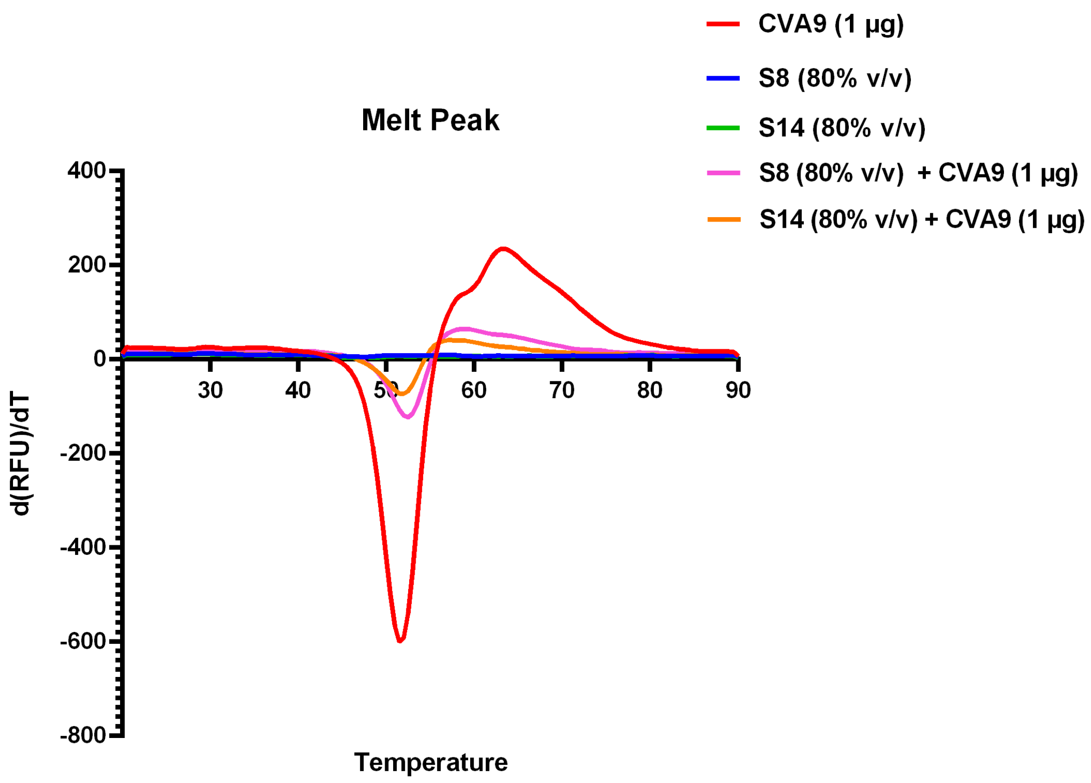

Supplementary Fig. S4. Melt peak Tm, plotted from the thermal assay. Selected hit ferments (S8, S14) have been pre-incubated with CVA9 for 1 h at 37 and then subjected to thermal assay (see Material and Methods for details). The melt peak is here plotted using the derivative of relative fluorescence units (RFU) as a function of temperature (d(RFU)/dT).

Supplementary Table S1. The identified metabolites from ferment of *Ganoderma* sp. strains 8, 11, 14 and 17. Confidence of the metabolite identification is expressed in Metabolomics Standards Initiative (MSI) value proposed by Sumner et al. [1].

ID	Suggested compound	Rt (mir	Ion identity	Measured i	Calculated ic	Elemental co the ion [a)]	Mass er	DBE	MSI [b)]	MS/MS fragmentation (Top 5)	Ref.
**1**	Terpenoid	1.85	$[M-H]^-$	549.08882	549.08859	$C_{24}H_{21}O_{15}$	0.413	14	4	111 (100), 79 (35), 211 (32), 437 (17)	
			$[M+H]^+$	551.10272	551.10315	$C_{24}H_{23}O_{15}$	–0.774			Not present	
**2**	Triterpenoid	2.84	$[M-H]^-$	627.26587	627.16583	$C_{30}H_{43}O_{14}$	0.065	9	4	583 (100), 127 (64), 212 (52), 88 (43), 71 (34)	
			$[M+H]^+$	629.27960	629.28038	$C_{30}H_{45}O_{14}$	–1.243			70 (100), 330 (17), 169 (16), 74 (11), 468 (10)	
**3**	Sesquiterpenoid	3.10	$[M-H]^-$	301.16620	301.16566	$C_{15}H_{25}O_6$	1.787	3	4	87 (100), 57 (25), 71 (17), 151 (15), 113 (15)	
**4**	Sesquiterpenoid	3.19	$[M-H]^-$	303.18176	303.18131	$C_{15}H_{27}O_6$	1.478	2	4	59 (100), 57 (10), 87 (10), 243 (7), 155 (6)	
			$[M+Na]^+$	327.17761	327.17781	$C_{15}H_{28}O_6Na$	–0.610			265 (100), 309 (34), 70 (22), 86 (14), 120 (10)	
**5**	Sesquiterpenoid	3.27	$[M-H]^-$	303.18182	303.18131	$C_{15}H_{27}O_6$	1.676	2	4	59 (100), 224 (71), 130 (64), 123 (40), 94 (32)	
**6**	Terpenoid	3.51	$[M-H]^-$	517.22968	517.22905	$C_{24}H_{37}O_{12}$	1.219	6	4	59 (100), 71 (72), 99 (65), 205 (41), 153 (41)	
**7**	7-(hydroxymethyl)-3a,4,8a-trimethyldecahydro-4,8-meth 2,5,6,7-tetraol or isomer	4.10	$[M-H]^-$	285.17105	285.17075	$C_{15}H_{25}O_5$	1.062	3	3	59 (100), 97 (9), 139 (9)	
			$[M-2H_2O+H]^+$	251.16409	251.16417	$C_{15}H_{23}O_3$	–0.323			67 (100), 109 (82), 81 (74), 71 (69), 133 (69)	
**8**	7-(hydroxymethyl)-3a,4,8a-trimethyldecahydro-4,8-meth 2,5,6,7-tetraol or isomer	4.30	$[M-H]^-$	285.17102	285.17075	$C_{15}H_{25}O_5$	0.957	3	3	87 (100), 71 (56), 57 (52), 241 (32), 101 (21)	
			$[M-2H_2O+H]^+$	251.16397	251.16417	$C_{15}H_{23}O_3$	–0.801			81 (100), 79 (53), 135 (53), 107 (49), 93 (39)	
			$[M+Na]^+$	309.16708	309.16725	$C_{15}H_{26}O_5Na$	–0.534			291 (100), 123 (24), 105 (20), 247 (20), 91 (19)	
**9**	7-(hydroxymethyl)-3a,4,8a-trimethyldecahydro-4,8-meth 2,5,7-triol or isomer	4.37	$[M-H]^-$	269.17617	269.17583	$C_{15}H_{25}O_4$	1.254	3	3	254 (100), 71 (79), 197 (33), 117 (18), 121 (17)	2
**10**		4.38	$[M-H]^-$	285.17072	285.17075	$C_{15}H_{25}O_5$	–0.095	3	3	87 (100), 57 (63), 71 (62), 101 (33), 241 (32)	
			$[M+Na]^+$	309.16711	309.16725	$C_{15}H_{26}O_5Na$	–0.437			291 (100), 247 (17), 203 (13), 91 (7), 105 (7)	

	7-(hydroxymethyl)-3a,4,8a-trimethyldecahydro-4,8-meth 2,5,6,7-tetraol or isomer		$[M-2H_2O+H]^+$	251.16411	251.16417	$C_{15}H_{23}O_3$	–0.243			81 (100), 107 (60), 79 (60), 135 (52), 93 (47)	
**11**	Hydroxyganoderic acid G der	4.48	$[M-H]^-$	549.30792	549.30691	$C_{30}H_{45}O_9$	1.846	8	3	83 (100), 531 (58), 97 (49), 107 (48), 269 (45)	3
			$[M-H_2O+H]^+$	533.31158	533.31089	$C_{30}H_{45}O_8$	1.285			70 (100), 91 (78), 105 (74), 81 (51), 79 (48)	
**12**	Hydroxypentadecanedioic aci	4.58	$[M-H]^-$	287.18613	287.1864	$C_{15}H_{27}O_5$	–0.930	2	3	59 (100), 151 (6), 57 (6), 85 (6), 285 (4)	
**13**	Hydroxyganoderic acid G der	4.63	$[M-H]^-$	549.30775	549.30691	$C_{30}H_{45}O_9$	1.536	8	3	69 (100), 113 (51), 131 (50), 101 (35), 83 (32)	
**14**	Ganoderic acid L or isomer	4.65	$[M-H]^-$	533.31294	533.31199	$C_{30}H_{45}O_8$	1.778	8	2	85 (100), 503 (30), 83 (29), 97 (21), 149 (13)	4
**15**	Triterpenoid	4.73	$[M-H]^-$	643.29614	643.29713	$C_{31}H_{47}O_{14}$	–1.538	8	3	110 (100), 625 (86), 96 (60), 242 (36), 577 (35)	5
			$[M+H]^+$	645.30970	645.31168	$C_{31}H_{49}O_{14}$	–3.072			86 (100), 74 (27), 514 (23), 88 (23), 69 (17)	
**16**	Hydroxyganoderic acid G der	4.79	$[M-H]^-$	549.30748	549.30691	$C_{30}H_{45}O_9$	1.045	8	3	517 (100), 83 (58), 318 (50), 518 (38), 505 (31)	3
**17**	Triterpenoid	4.82	$[M-H]^-$	577.26617	577.26544	$C_{30}H_{41}O_{11}$	1.273	10	3	487 (100), 83 (21), 107 (16), 259 (11), 325 (10)	
**18**	Soyasaponin type compound	5.01	$[M-H]^-$	1119.52313	1119.52289	$C_{53}H_{83}O_{25}$	0.214	12	3	85 (100), 113 (74), 101 (70), 99 (59), 89 (38)	6
			$[M+H]^+$	1121.53802	1121.53744	$C_{53}H_{85}O_{25}$	0.513			453 (100), 435 (83), 85 (66), 141 (45), 105 (44)	
**19**	Soyasaponin A2	5.03	$[M-H]^-$	1105.54410	1105.54363	$C_{53}H_{85}O_{24}$	0.428	11	2	85 (100), 113 (72), 101 (71), 99 (53), 95 (37)	
			$[M+H]^+$	1107.55744	1107.55818	$C_{53}H_{87}O_{24}$	–0.688			439 (100), 421 (78), 85 (53), 95 (46), 141 (45)	
**20**	20-Hydroxylganoderic acid G	5.05	$[M-H]^-$	547.29217	547.29126	$C_{30}H_{43}O_9$	1.670	9	3	517 (100), 115 (97), 83 (77), 171 (77), 433 (76)	3
			$[M-H_2O+H]^+$	531.29474	531.29524	$C_{30}H_{43}O_8$	–0.950			70 (100), 91 (94), 105 (81), 93 (69), 119 (67)	
**21**	Soyasaponin type compound	5.10	$[M-H]^-$	1089.54889	1089.54871	$C_{53}H_{85}O_{23}$	0.164	11	3	85 (100), 113 (71), 99 (70), 87 (56), 101 (49)	7
			$[M+H]^+$	1091.56348	1091.56327	$C_{53}H_{87}O_{23}$	0.197			439 (100), 421 (75), 85 (69), 95 (47), 141 (35)	
**22**	Diterpenoid	5.10	$[M-H]^-$	369.22855	369.22826	$C_{20}H_{33}O_6$	0.780	4	3	87 (100), 57 (65), 59 (44), 83 (23), 99 (21)	
**23**	Diterpenoid	5.19	$[M-H]^-$	383.20748	383.20753	$C_{20}H_{31}O_7$	–0.121	5	3	339 (100), 85 (57), 87 (31), 57 (25), 71 (24)	
**24**	Ganoderic acid L or isomer	5.28	$[M-H]^-$	533.31230	533.31199	$C_{30}H_{45}O_8$	0.578	8	2	97 (100), 83 (78), 171 (54), 413 (33), 355 (30)	4
**25**	Soyasaponin type compound	5.43	$[M-H]^-$	1103.52800	1103.52798	$C_{53}H_{83}O_{24}$	0.021	12	3	85 (100), 113 (69), 101 (64), 99 (51), 89 (40)	8
			$[M+H]^+$	1105.54038	1105.54253	$C_{53}H_{85}O_{24}$	–1.944			587 (100), 419 (87), 437 (85), 85 (72), 141 (55)	
**26**	Ganoderic acid G isomer	5.44	$[M-H]^-$	531.29661	531.29634	$C_{30}H_{43}O_8$	0.505	9	2	165 (100), 57 (76), 501 (65), 107 (56), 121 (51)	9
			$[M-H_2O+H]^+$	515.29994	515.30033	$C_{30}H_{43}O_7$	–0.757			91 (100), 105 (83), 70 (82), 93 (63), 95 (59)	

			$[M+H]^+$	533.30997	533.31089	$C_{30}H_{45}O_8$	–1.734			515 (100), 91 (96), 105 (88), 369 (81), 145 (73)	
**27**	Triterpenoid	5.60	$[M-H]^-$	535.32764	535.32764	$C_{30}H_{47}O_8$	–0.003	7	3	83 (100), 97 (74), 415 (71), 455 (47), 107 (37)	
**28**	Platycodigenin isomer	5.65	$[M-H]^-$	519.33286	519.33273	$C_{30}H_{47}O_7$	0.256	7	3	85 (100), 97 (18), 415 (18), 107 (11), 69 (9)	
**29**	Ganoderic acid L or isomer	5.67	$[M-H]^-$	533.31250	533.31199	$C_{30}H_{45}O_8$	0.953	8	2	85 (100), 135 (66), 83 (47), 109 (47),73 (36)	4
			$[M-H_2O+H]^+$	517.31518	517.31598	$C_{30}H_{45}O_7$	–1.547			105 (100), 93 (93), 499 (89), 91 (88), 67 (80)	
			$[M+H]^+$	535.32547	535.32654	$C_{30}H_{47}O_8$	–2.008			517 (100), 518 (36), 105 (33), 481 (30), 91 (29)	
**30**	Ganoderic acid C2 or isomer	5.80	$[M-H]^-$	517.31655	517.31708	$C_{30}H_{45}O_7$	–1.019	8	2	73 (100), 109 (56), 137 (48), 165 (46), 83 (40)	10
			$[M-3H_2O+H]^+$	465.29898	465.29994	$C_{30}H_{41}O_4$	–2.055			81 (100), 105 (73), 123 (73), 401 (48), 93 (48)	
			$[M-2H_2O+H]^+$	483.30903	483.3105	$C_{30}H_{43}O_5$	–3.043			105 (100), 465 (99), 81 (96), 419 (94), 401 (79)	
**31**	Triterpenoid derivative	5.85	$[M-H]^-$	521.31230	521.31199	$C_{29}H_{45}O_8$	0.592	7	3	101 (100), 71 (71), 57 (46), 109 (43), 221 (40)	
**32**	Triterpenoid	5.90	$[M-H]^-$	535.32726	535.32764	$C_{30}H_{47}O_8$	–0.713	7	3	521 (100), 522 (36), 101 (11, 475 (11), 523 (8)	
			$[M-2H_2O+H]^+$	501.31933	501.32107	$C_{30}H_{45}O_6$	–3.462			105 (100), 91 (84), 119 (84), 95 (78), 93 (77)	
**33**	Triterpenoid	5.96	$[M-H]^-$	535.32745	535.32764	$C_{30}H_{47}O_8$	–0.358	7	3	73 (100), 85 (66), 59 (33), 109 (33), 57 (27)	
			$[M-2H_2O+H]^+$	501.31981	501.32107	$C_{30}H_{45}O_6$	–2.504			437 (100), 105 (90), 483 (49), 91 (41), 93 (40)	
			$[M-H_2O+H]^+$	519.33027	519.33163	$C_{30}H_{47}O_7$	–2.619			501 (100), 105 (60), 483 (59), 119 (51), 91 (47)	
**34**	Triterpenoid	6.07	$[M-H]^-$	535.32780	535.32764	$C_{30}H_{47}O_8$	0.296	7	3	73 (100), 85 (71), 115 (48), 99 (39), 59 (37)	
			$[M-2H_2O+H]^+$	501.31982	501.32107	$C_{30}H_{45}O_6$	–2.485			437 (100), 501 (88), 105 (83), 483 (50), 73 (41)	
			$[M-H_2O+H]^+$	519.33053	519.33163	$C_{30}H_{47}O_7$	–2.119			89 (100), 501 (91), 105 (87), 465 (80), 93 (78)	
**35**	Ganoderic acid A or isomer	6.13	$[M-H]^-$	515.30176	515.30143	$C_{30}H_{43}O_7$	0.646	9	2	271 (100), 83 (97), 85 (82), 99 (68), 97 (59)	10
			$[M+H]^+$	517.31506	517.31598	$C_{30}H_{45}O_7$	–1.779			499 (100), 91 (80), 463 (76), 93 (75), 105 (72)	
**36**	Ganoderic acid L or isomer	6.19	$[M-H]^-$	533.31250	533.31199	$C_{30}H_{45}O_8$	0.953	8	2	73 (100), 85 (44), 135 (37), 149 (34), 83 (33)	4
			$[M-H_2O+H]^+$	517.31451	517.31598	$C_{30}H_{45}O_7$	–2.842			499 (100), 463 (91), 343 (76), 93 (67), 67 (64)	
**37**	Ganoderic acid A or isomer	6.27	$[M-H]^-$	515.30182	515.30143	$C_{30}H_{43}O_7$	0.763	9	2	83 (100), 153 (39), 497 (19), 71 (18), 485 (18)	10
			$[M+H]^+$	517.31592	517.31598	$C_{30}H_{45}O_7$	–0.116			499 (100), 105 (90), 91 (85), 107 (71), 119 (71)	
**38**	Soyasaponin Bd or isomer [c)]	6.29	$[M-H]^-$	955.49080	955.4908	$C_{48}H_{75}O_{19}$	0.625	11	3	71 (100), 85 (72), 99 (47), 87 (43), 75 (42)	11
			$[M+H]^+$	957.50336	957.50536	$C_{48}H_{77}O_{19}$	–2.085			455 (100), 473 (37), 456 (31, 85 (29), 437 (29)	

			$[M+Na]^+$	979.48444	979.4873	$C_{48}H_{76}O_{19}Na$	–2.921			Not present	
**39**	Platycodigenin isomer	6.30	$[M-H]^-$	519.33278	519.33273	$C_{30}H_{47}O_7$	0.102	7	3	85 (100), 97 (18), 415 (18), 107 (11), 287 (7)	12
			$[M-2H_2O+H]^+$	485.32449	485.32615	$C_{30}H_{45}O_5$	–3.422			95 (100), 93 (68), 91 (63), 67 (62), 107 (62)	
**40**	Ganoderic acid C2 or isomer	6.36	$[M-H]^-$	517.31740	517.31708	$C_{30}H_{45}O_7$	0.624	8	2	415 (100), 455 (62), 416 (35), 83 (30), 289 (29)	10
			$[M-H_2O+H]^+$	501.31995	501.32107	$C_{30}H_{45}O_6$	–3.023			73 (100), 241 (83), 243 (33), 57 (21), 242 (17)	
			$[M+H]^+$	519.33008	519.33163	$C_{30}H_{47}O_7$	–2.985			501 (100), 502 (36), 215 (33), 105 (28), 91 (27)	
			$[M+Na]^+$	541.31219	541.31357	$C_{30}H_{46}O_7Na$	–2.558			Not present	
**41**	Triterpenoid derivative	6.39	$[M-H]^-$	505.31767	505.31708	$C_{29}H_{45}O_7$	1.174	7	3	101 (100), 71 (52), 58 (45), 59 (44), 109 (28)	
**42**	Pinellic acid	6.45	$[M-H]^-$	329.23352	329.23335	$C_{18}H_{33}O_5$	0.524	2	3	171 (100), 211 (50), 139 (46), 99 (32), 127 (23)	13
			$[M+Na]^+$	353.22882	353.22985	$C_{18}H_{34}O_5Na$	–2.903			120 (100), 227 (96), 181 (87), 70 (65), 84 (48)	
**43**	Triterpenoid derivative	6.45	$[M-H]^-$	505.31762	505.31708	$C_{29}H_{45}O_7$	1.075	7	3	101 (100), 71 (65), 59 (46), 58 (26), 83 (25)	
**44**	Cucurbitacin IIa	6.48	$[M-H]^-$	561.34375	561.34329	$C_{32}H_{49}O_8$	0.816	8	3	85 (100), 519 (45), 501 (28), 59 (22), 415 (21)	
**45**	Ganoderic acid N or isomer	6.49	$[M-H]^-$	529.28089	529.28069	$C_{30}H_{41}O_8$	0.375	10	2	437 (100), 308 (59), 99 (50), 293 (43), 309 (38)	14
			$[M+H]^+$	531.29547	531.29524	$C_{30}H_{43}O_8$	0.424			69 (100), 495 (89), 91 (72), 105 (59), 93 (49)	
**46**	Ganoderic acid C2 or isomer	6.53	$[M-H]^-$	517.31744	517.31708	$C_{30}H_{45}O_7$	0.702	8	2	83 (100), 69 (69), 57 (51), 73 (34), 113 (27)	10
			$[M+H]^+$	519.33075	519.33163	$C_{30}H_{47}O_7$	–1.695			501 (100), 465 (39), 93 (38), 105 (38), 91 (37)	
			$[M-H_2O+H]^+$	501.31985	501.32107	$C_{30}H_{45}O_6$	–2.425			105 (100), 91 (91), 483 (90), 119 (81) 95 (70)	
**47**	Triterpenoid	6.58	$[M-H]^-$	521.34854	521.34838	$C_{30}H_{49}O_7$	0.313	6	3	73 (100), 99 (68), 241 (17), 243 (17), 101 (12)	
			$[M-H_2O+H]^+$	505.35144	505.35237	$C_{30}H_{49}O_6$	–1.832			487 (100), 488 (34), 81 (29), 95 (25), 69 (19)	
**48**	Ganoderic acid C2 or isomer	6.64	$[M-H]^-$	517.31736	517.31708	$C_{30}H_{45}O_7$	0.547	8	2	115 (100), 97 (77), 99 (65), 73 (61), 83 (56)	10
**49**	Cucurbitacin IIb isomer	6.69	[M-H]-	519.33275	519.33273	$C_{30}H_{47}O_7$	0.044	7	3	73 (100), 57 (41), 123 (23), 247 (18), 137 (13)	12
**50**	Ganoderic acid C2 or isomer	6.73	$[M-H]^-$	517.31671	517.31708	$C_{30}H_{45}O_7$	–0.709	8	2	69 (100), 73 (78), 57 (58), 113 (57), 85 (50)	10
			$[M-H_2O+H]^+$	501.32010	501.32107	$C_{30}H_{45}O_6$	–1.926			483 (100), 59 (72), 105 (59), 91 (55), 93 (50)	
			$[M+H]^+$	519.33029	519.33163	$C_{30}H_{47}O_7$	–2.581			501 (100), 483 (92), 465 (45), 105 (45), 201 (44)	
**51**	Soyasaponin Bd or isomer [c)]	6.77	$[M-H]^-$	955.49126	955.4908	$C_{48}H_{75}O_{19}$	0.478	11	3	71 (100), 85 (90), 99 (61), 87 (53), 75 (38)	11
			$[M+H]^+$	957.50342	957.50536	$C_{48}H_{77}O_{19}$	–2.022			Not present	

**52**	Ganoderic acid A or isomer	6.78	$[M-H]^-$	515.30150	515.30143	$C_{30}H_{43}O_7$	0.142	9	2	413 (100), 97 (85), 453 (71), 83 (40), 414 (37)	[10]
			$[M+H]^+$	517.31519	517.31598	$C_{30}H_{45}O_7$	–1.527			499 (100), 353 (82), 215 (75), 105 (68), 91 (63)	
			$[M-H_2O+H]^+$	499.30453	499.30542	$C_{30}H_{43}O_6$	–1.773			105 (100), 91 (96), 59 (94), 93 (82), 119 (73)	
**53**	Ganoderic acid C2 or isomer	6.87	$[M-H]^-$	517.31747	517.31708	$C_{30}H_{45}O_7$	0.760	8	2	73 (100), 85 (89), 499 (40), 97 (22), 109 (20)	[10]
			$[M+H]^+$	519.33130	519.33163	$C_{30}H_{47}O_7$	–0.636			105 (100), 91 (85), 501 (76), 93 (74), 119 (74)	
**54**	3-O-Acetyl ganoderic acid A	6.90	$[M-H]^-$	557.31250	557.31199	$C_{32}H_{45}O_8$	0.912	10	2	519 (100), 520 (67), 73 (16), 521 (15), 101 (10)	[15]
			$[M-H_2O+H]^+$	541.31311	541.31598	$C_{32}H_{45}O_7$	–5.302			463 (100), 93 (75), 481 (70), 91 (68), 95 (67)	
**55**	Cucurbitacin IIb isomer	6.92	$[M-H]^-$	519.3321	519.33273	$C_{30}H_{47}O_7$	–1.207	7	3	73 (100), 101 (49), 221 (26), 71 (20), 501 (19)	[12]
			$[M-2H_2O+H]^+$	485.32382	485.32615	$C_{30}H_{45}O_5$	–4.803			421 (100), 105 (71), 422 (34), 81 (16), 439 (16)	
			$[M-H_2O+H]^+$	503.33378	503.33672	$C_{30}H_{47}O_6$	–5.832			485 (100), 486 (36), 105 (20), 81 (19), 119 (17)	
**56**	Cucurbitacin IIb isomer	6.99	$[M-H]^-$	519.33237	519.33273	$C_{30}H_{47}O_7$	–0.687	7	3	73 (100), 101 (53), 71 (29), 221 (26), 109 (22)	[12]
			$[M-2H_2O+H]^+$	485.32404	485.32615	$C_{30}H_{45}O_5$	–4.350			421 (100), 105 (71), 422 (36), 81 (15), 145 (15)	
			$[M-H_2O+H]^+$	503.33376	503.33672	$C_{30}H_{47}O_6$	–5.872			485 (100), 486 (34), 105 (21), 81 (20), 119 (16)	
**57**	Ganoderic acid C2 or isomer	7.05	$[M-H]^-$	517.31685	517.31708	$C_{30}H_{45}O_7$	–0.439	8	2	73 (100), 57 (30), 123 (24), 109 (19), 97 (11)	[10]
			$[M-H_2O+H]^+$	501.31929	501.32107	$C_{30}H_{45}O_6$	–3.542			437 (100), 105 (57), 483 (57), 91 (39), 119 (38)	
**58**	Cucurbitacin IIb isomer	7.11	$[M-H]^-$	519.33289	519.33273	$C_{30}H_{47}O_7$	0.314	7	3	73 (100), 109 (38), 71 (26), 135 (26), 319 (25)	[12]
**59**	3-O-Acetyl ganoderic acid A	7.17	$[M-H]^-$	557.31165	557.31199	$C_{32}H_{45}O_8$	–0.613	10	2	59 (100), 85 (25), 423 (19), 310 (15), 295 (8)	[15]
			$[M+H]^+$	559.32471	559.32654	$C_{32}H_{47}O_8$	–3.280			Not present	
**60**	3-O-Acetyl ganoderic acid A	7.20	$[M-H]^-$	557.31286	557.31199	$C_{32}H_{45}O_8$	1.558	10	2	59 (100), 85 (12), 517 (10), 311 (9), 423 (8)	[15]
**61**	Ganorbifromin C	7.25	$[M-H]^-$	503.33818	503.33781	$C_{30}H_{47}O_6$	0.730	7	3	73 (100), 99 (70), 243 (21), 241 (17), 57 (10)	[16]
**62**	Ganolucidic acid B or isomer	7.28	$[M-H]^-$	501.32291	501.32216	$C_{30}H_{45}O_6$	1.491	8	2	85 (100), 415 (70), 99 (34), 92 (22), 183 (19)	[17]
			$[M-H_2O+H]^+$	485.32523	485.32615	$C_{30}H_{45}O_5$	–1.898			105 (100), 421 (61), 95 (58), 93 (55), 91 (53)	
			$[M+H]^+$	503.33563	503.33672	$C_{30}H_{47}O_6$	–2.157			485 (100), 449 (36), 486 (35), 93 (32), 95 (31)	
**63**	Unknown compound	7.35	$[M-H]^-$	527.24361	527.24391	$C_{33}H_{35}O_6$	–0.573	16	4	273 (100), 143 (92), 255 (52), 99 (44), 85 (43)	
**64**	Hovenidulcigenin A or isome	7.48	$[M-H]^-$	543.33344	543.33273	$C_{32}H_{47}O_7$	1.312	9	2	501 (100), 59 (51), 85 (41), 502 (31), 415 (31)	
**65**	Dehydrosoyasaponin I [c)]	7.48	$[M-H]^-$	939.49738	939.49589	$C_{48}H_{75}O_{18}$	1.588	11	3	71 (100), 85 (68), 75 (47), 99 (47), 87 (41)	

**66**	Ganoderic acid C2 or isomer	7.52	[M-H]$^-$	517.31720	517.31708	$C_{30}H_{45}O_7$	0.238	8	2	135 (100), 109 (82), 319 (53), 101 (41), 99 (22)	[10]
**67**	Soyasaponin I (*syn.* soyasapo	7.52	[M-H]$^-$	941.51270	941.51154	$C_{48}H_{77}O_{18}$	1.234	10	2	71 (100), 85 (75), 99 (47), 75 (42), 113 (42)	[18]
**68**	3-O-Acetyl ganoderic acid A	7.60	[M-H]$^-$	557.31268	557.31199	$C_{32}H_{45}O_8$	1.235	10	2	515 (100), 59 (60), 516 (29), 99 (25), 185 (18)	[15]
**69**	Ganolucidic acid A	7.62	[M-H]$^-$	499.30676	499.30651	$C_{30}H_{43}O_6$	0.496	9	2	83 (100), 97 (68), 85 (43), 57 (30), 255 (23)	[17]
**70**	Ganolucidic acid B or isomer	7.65	[M-H]$^-$	501.32247	501.32216	$C_{30}H_{45}O_6$	0.614	8	2	73 (100), 99 (75), 57 (31), 287 (26), 83 (22)	[17]
			[M+H]$^+$	503.33392	503.33672	$C_{30}H_{47}O_6$	–5.554			485 (100), 105 (72), 467 (72), 449 (64), 67 (63)	
**71**	Hovenidulcigenin A or isome	7.68	[M-H]$^-$	543.33293	543.33273	$C_{32}H_{47}O_7$	0.374	9	2	59 (100), 501 (58), 483 (47), 85 (30), 502 (16)	
**72**	3-O-Acetyl ganoderic acid A	7.70	[M-H]$^-$	557.31232	557.31199	$C_{32}H_{45}O_8$	0.589	10	2	59 (100), 453 (42), 513 (18), 99 (17), 454 (12)	[15]

DBE = double bond equivalent

[a] The elemental composition of the metabolites was restricted to contain C, H and O, except for positive ionization also Na adducts were included

[b] MSI scale range: 1-4; level 1 = rigorously identified compound, e.g. with authentic chemical standard; level 2 = putative annotated compound, meaning in here that identification is based upon mass spectral similarity with public/commercial spectral libraries; level 3 = putatively characterized compound class, meaning in here that identification is based upon mass spectral similarity to known compounds of a chemical class; level 4 = unknown compound, meaning in here that metabolite is differentiated based upon mass spectral data

[c] Compounds **38**, **51**, **65**, and **67** are present in the culture medium as well, and therefore presumed to originate from the peptone added to the culture medium

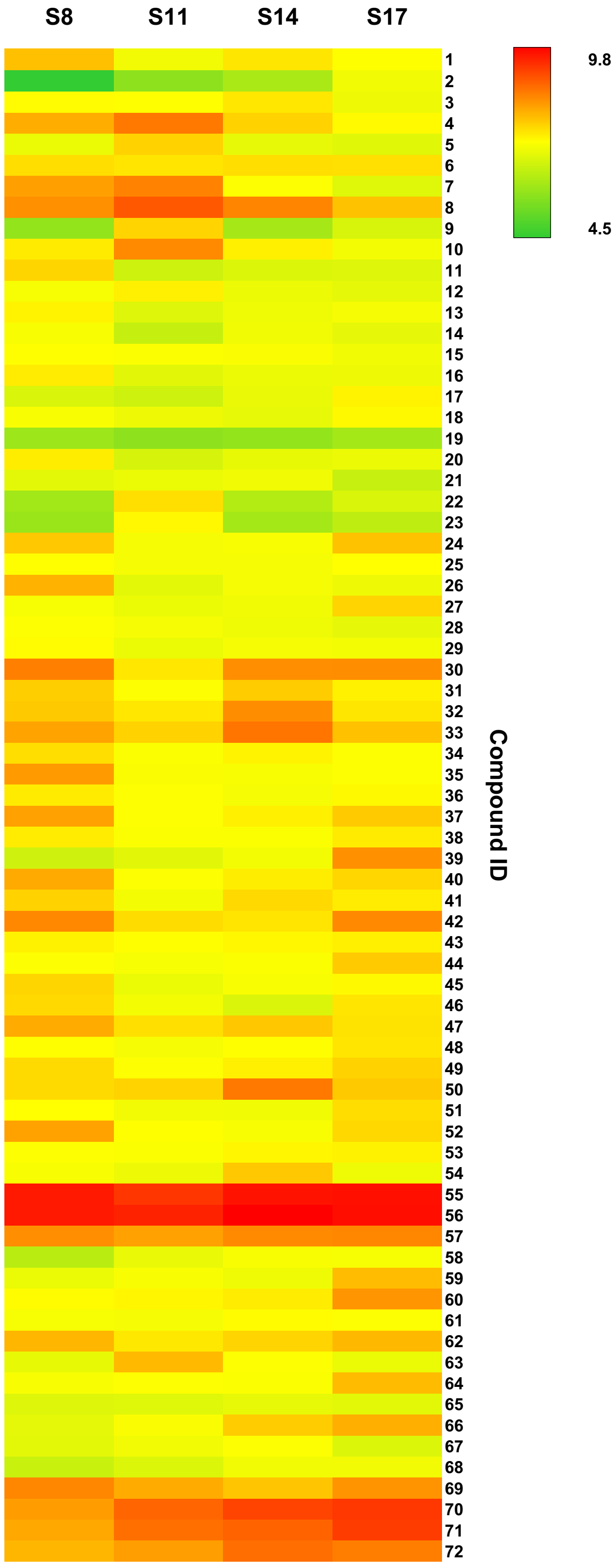

Supplementary Figure S5. The metabolite content of *Ganoderma* sp. strains 8, 11, 14 and 17 ferments displayed as a heatmap, with values expressed as the Log10-transformed, compound-specific peak areas of detected ions under negative ion mode varying from 4.5 to 9.8. The compound numbers correspond to those listed in Supplementary Table S1.

A

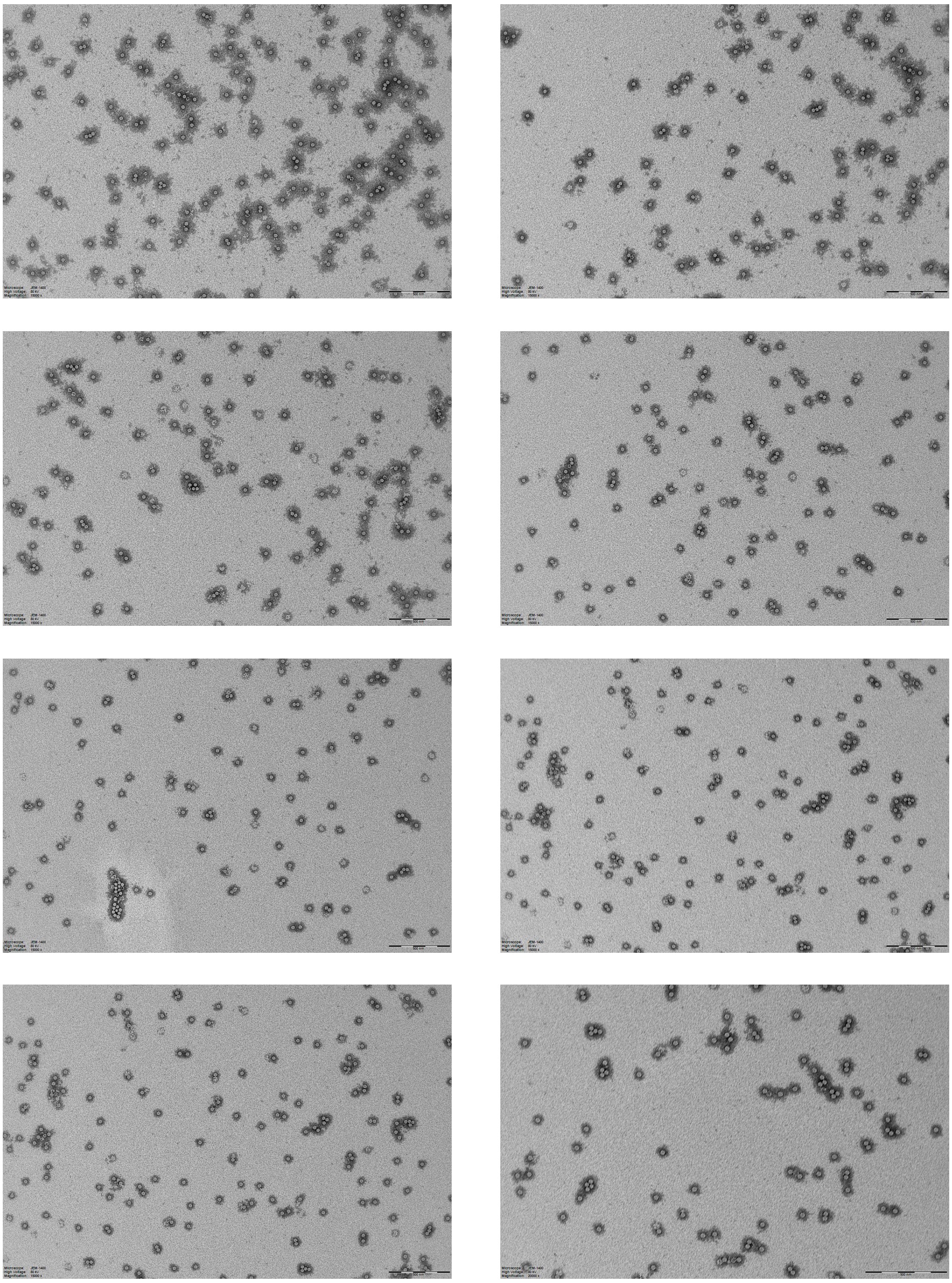

B

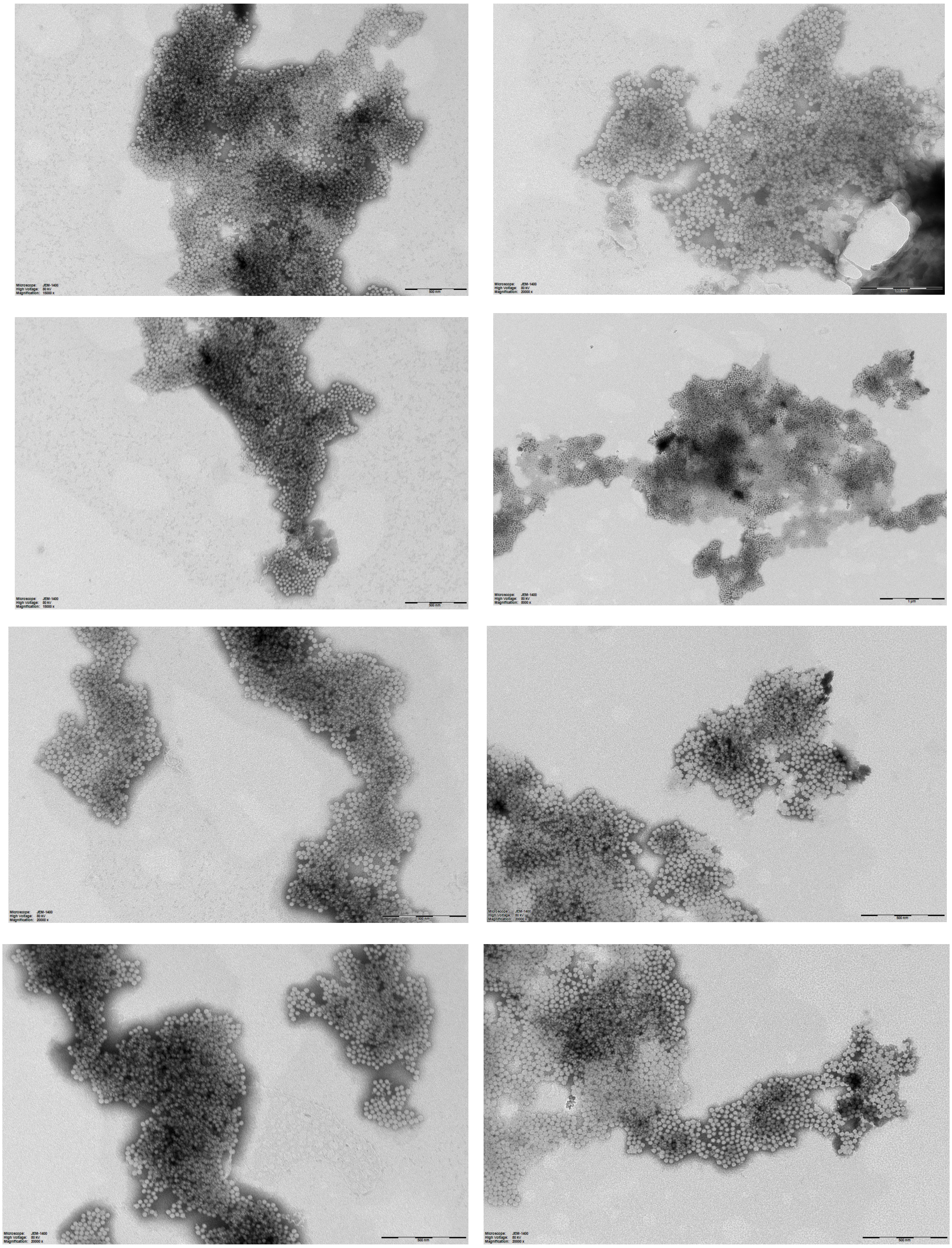

Supplementary Fig. S6. TEM images of CVA9 treated with control buffer or with S14 for 1 h at 37C. (A) Several images of CVA9 treated with control buffer PBS-MgCl2 and in (B) several areas of S14 -treated CVA9. The scale bars in the images are between 500 nm and 1μm.

References

1. Sumner, L. W. *et al.* Proposed minimum reporting standards for chemical analysis Chemical Analysis Working Group (CAWG) Metabolomics Standards Initiative (MSI). *Metabolomics* **3**, 211–221 (2007).
2. Binh, P. T., Descoutures, D., Dang, N. H., Nguyen, N. P. D. & Dat, N. T. A New Cytotoxic Gymnomitrane Sesquiterpene from Ganoderma lucidum Fruiting Bodies. *Nat Prod Commun* **10**, 1911–1912 (2015).
3. Ma, J. *et al.* New lanostanoids from the mushroom Ganoderma lucidum. *J Nat Prod* **65**, 72–75 (2002).
4. Nishitoba, T., Oda, K., Sato, H. & Sakamura, S. Novel Triterpenoids from the Fungus Ganoderma lucidum. *Agricultural and Biological Chemistry* **52**, 367–372 (1988).
5. Lee, I. *et al.* Lanostane triterpenes from the fruiting bodies of Ganoderma lucidum and their inhibitory effects on adipocyte differentiation in 3T3-L1 Cells. *J Nat Prod* **73**, 172–176 (2010).
6. Witkowska, H. E., Bialy, Z., Jurzysta, M. & Waller, G. R. Analysis of Saponin Mixtures from Alfalfa (*Medicago Sativa L.*) Roots using Mass Spectrometry with MALDI Techniques. *Natural Product Communications* **3**, 1934578X0800300 (2008).
7. Tava, A., Pecetti, L., Romani, M., Mella, M. & Avato, P. Triterpenoid Glycosides from the Leaves of Two Cultivars of Medicago polymorpha L. *J. Agric. Food Chem.* **59**, 6142–6149 (2011).
8. van Dooren, I. *et al.* Saponins and Flavonoids from an Infusion of Herniaria hirsuta. *Planta Med* **82**, 1576–1583 (2016).

9. Qi, Y., Zhao, L. & Sun, H. H. Development of a Rapid and Confirmatory Method to Identify Ganoderic Acids in Ganoderma Mushrooms. *Front Pharmacol* **3**, 85 (2012).
10. Liang, C. *et al.* Review of the molecular mechanisms of Ganoderma lucidum triterpenoids: Ganoderic acids A, C2, D, F, DM, X and Y. *Eur J Med Chem* **174**, 130–141 (2019).
11. Choi, J. N. *et al.* Metabolomics revealed novel isoflavones and optimal cultivation time of Cordyceps militaris fermentation. *J Agric Food Chem* **58**, 4258–4267 (2010).
12. Zhang, X. *et al.* Hepatoprotective Activity of Ethanol Extract of Rice Solid-State Fermentation of Ganoderma tsugae against CCl4-Induced Acute Liver Injury in Mice. *Molecules* **27**, 5347 (2022).
13. Wahba, A. E., El-Sayed, A. K. A., El-Falal, A. A. & Soliman, E. M. New antimalarial lanostane triterpenes from a new isolate of Egyptian Ganoderma species. *Med Chem Res* **28**, 2246–2251 (2019).
14. Nishitoba, T., Sato, H., Shirasu, S. & Sakamura, S. Evidence on the Strain-specific Terpenoid Pattern of Ganoderma lucidum. *Agricultural and Biological Chemistry* **50**, 2151–2154 (1986).
15. Chang, T.-S., Ko, H.-H., Wang, T.-Y., Lee, C.-H. & Wu, J.-Y. Biotransformation of Ganoderic Acid A to 3-O-Acetyl Ganoderic Acid A by Soil-isolated Streptomyces sp. *Fermentation* **4**, 101 (2018).
16. Isaka, M., Chinthanom, P., Kongthong, S., Srichomthong, K. & Choeyklin, R. Lanostane triterpenes from cultures of the Basidiomycete Ganoderma orbiforme BCC 22324. *Phytochemistry* **87**, 133–139 (2013).
17. Kikuchi, T., Matsuda, S., Murai, Y. & Ogita, Z. Ganoderic Acid G and I and Ganolucidic Acid a and B, New Triterpenoids from Ganoderma Lucidum. *Chemical & Pharmaceutical Bulletin* **33**, 2628–2631 (1985).
18. Lee, S.-Y., Kim, J.-S., Shim, S.-H. & Kang, S.-S. Soyasaponins from Soybean Flour Medium for the Liquid Culture of Ganoderma applanatum. *Bulletin of the Korean Chemical Society* **32**, 3650–3654 (2011).
